# *EPAS1* knockdown is associated with cell cycle and DNA replication programs and MYC/E2F-related signatures in hemangioma endothelial cells

**DOI:** 10.1371/journal.pone.0354272

**Published:** 2026-07-24

**Authors:** Zheren Su, Luying Wang, Ruixue Zhao, Zhiyu Li, Jing Li, Jianhai Bi, Ran Huo

**Affiliations:** 1 Department of Plastic and Aesthetic Surgery, Shandong Provincial Hospital, Cheeloo College of Medicine, Shandong University, Jinan, Shandong, China; 2 Department of Plastic and Aesthetic Surgery, Shandong Provincial Hospital Affiliated to Shandong First Medical University, Jinan, Shandong, China; Tianjin Medical University Cancer Institute and Hospital: Tianjin Medical University Cancer Institute & Hospital, CHINA

## Abstract

Infantile hemangioma (IH) is the most common benign tumor of infancy. Hypoxia and activation of hypoxia-inducible factor (HIF) signaling have been proposed to contribute to IH pathogenesis, yet the role of endothelial PAS domain-containing protein 1 (*EPAS1*), which encodes hypoxia-inducible factor-2α (HIF-2α), in hemangioma endothelial cells (HemECs) remains less well characterized. Here, we investigated HIF-2α in primary HemECs using a pharmacological inhibitor (PT-2399) and shRNA-mediated *EPAS1* knockdown under normoxic and hypoxic conditions. In hypoxic cultures, PT-2399 treatment was associated with reduced migration and invasion and with a reduction in junction number in tube formation assays; at the selected dose, PT-2399 did not significantly reduce cell viability. By contrast, *EPAS1* knockdown was associated with reduced proliferative capacity and altered cell cycle distribution, together with enrichment of DNA replication/cell cycle-related transcriptional programs, negative enrichment of MYC- and E2F-related gene sets, and directionally consistent protein level changes in selected regulators. *EPAS1* knockdown-associated phenotypic trends were broadly similar under normoxia and hypoxia, with no clear evidence that hypoxic stimulation enhanced the magnitude of these changes. In IH tissue transcriptomic data, Egl-9 family hypoxia-inducible factor 3 (*EGLN3*) showed reduced expression, consistent with a testable hypothesis that hypoxia-independent mechanisms may contribute to maintenance of HIF-2α activity in this context. This study is limited by the use of HemECs derived from a single IH specimen and by the absence of on-target validation; accordingly, the findings should be interpreted as exploratory and hypothesis-generating.

## Introduction

Infantile hemangioma (IH) is the most common benign tumor of infancy, characterized by a phase of rapid proliferation followed by gradual spontaneous involution [1]. Since propranolol was discovered as an effective treatment for IH, it has been widely adopted in clinical practice [2]. However, the mechanisms underlying IH development, as well as the precise modes of action of propranolol, remain incompletely understood.

The prevailing view of IH pathogenesis emphasizes the role of hypoxia-associated signaling, in which hypoxic stress is thought to promote the expression of angiogenic factors, such as vascular endothelial growth factor (VEGF), through activation of the hypoxia-inducible factor (HIF) pathway [3,4]. Under hypoxic conditions, HIF activity is regulated primarily through stabilization of hypoxia-inducible factor-1α (HIF-1α) and hypoxia-inducible factor-2α (HIF-2α), the latter encoded by endothelial PAS domain-containing protein 1 (*EPAS1*). These oxygen-regulated α subunits function by heterodimerizing with hypoxia-inducible factor-1β (HIF-1β), also known as aryl hydrocarbon receptor nuclear translocator (ARNT), to regulate transcription [5].

Although both HIF-1α and HIF-2α are central components of the HIF pathway, accumulating evidence indicates that these factors can exert distinct, context-dependent, and occasionally opposing biological effects, as exemplified in clear cell renal cell carcinoma (ccRCC) [6].

In contrast to the extensive investigation of HIF-1α in infantile hemangioma, the role of HIF-2α has received comparatively limited attention. HIF-1α is frequently described as a regulator of the proliferation of hemangioma endothelial cells (HemECs) and has been proposed as a molecular target of propranolol [2,7]. By comparison, studies specifically addressing HIF-2α in IH remain scarce.

To date, only a small number of reports have examined HIF-2α in this context. An immunohistochemical study by Giatromanolaki et al. reported positive HIF-2α staining in all examined IH samples, whereas HIF-1α was undetectable [8]. More recently, Gomez-Acevedo et al. performed an integrative transcriptomic analysis and identified *EPAS1* among a subset of genes showing differential expression across IH phases and responsiveness to propranolol exposure [9]. Together, these observations indicate that HIF-2α is consistently detectable in IH tissues and transcriptionally dynamic during disease progression, highlighting the need for further functional characterization of HIF-2α in IH.

Beyond the potential involvement of HIF-2α, the contribution of hypoxia to IH pathogenesis remains incompletely defined. Although clinical associations such as prematurity and activation of hypoxia-responsive signaling pathways have been interpreted as indirect evidence supporting hypoxia-related mechanisms [2,3,7], direct functional evidence demonstrating sustained hypoxic stress within IH tissues is limited.

Activation of hypoxia-inducible signaling can occur independently of reduced oxygen availability. One such scenario is pseudohypoxia, in which hypoxia-responsive transcriptional programs are engaged under normoxic conditions [10]. This phenomenon has been well documented in ccRCC, where disruption of oxygen-sensing machinery is associated with constitutive HIF activity [11]. These observations raise the possibility that hypoxia-independent regulation of HIF-related pathways may also be relevant in other pathological contexts, including IH.

In this study, we investigated the role of HIF-2α in HemECs. Using a pharmacological inhibitor and shRNA-mediated *EPAS1* knockdown, we assessed how HIF-2α perturbation influences HemEC phenotypes under normoxic and hypoxic conditions. This design allowed us to examine HIF-2α-associated phenotypes across oxygen conditions and to compare whether the phenotypic patterns of *EPAS1* knockdown differ between normoxia and hypoxia. To define the molecular features associated with *EPAS1* knockdown, we performed transcriptomic profiling to identify gene expression changes and pathway level signatures associated with *EPAS1* knockdown in HemECs. We further performed protein level analyses to examine selected candidate regulators highlighted by the transcriptomic profiles. Collectively, this study integrates functional perturbation with molecular profiling to clarify how HIF-2α may contribute to HemEC biology in the context of IH.

## Results

### Characterization of primary HemECs

**Fig 1:**
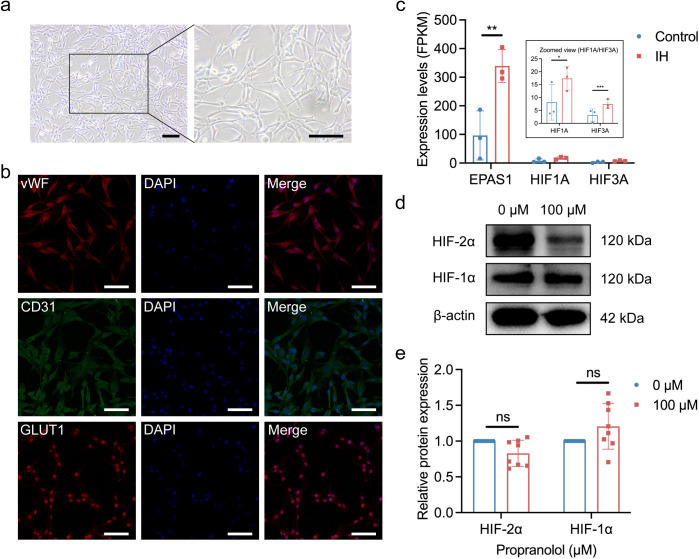
Characterization of primary HemECs and HIF-related expression in IH. (a) Representative light microscopy images showing the morphology of primary HemECs. Scale bars = 200 μm. (b) Immunofluorescence staining of primary HemECs for vWF, CD31, and GLUT1. Scale bars = 100 μm. Original immunofluorescence images are presented in S9 Fig in S1 File. (c) Bulk RNA-seq analysis of IH tissues showing mRNA expression levels of *EPAS1*, *HIF1A*, and *HIF3A*. Due to the substantially higher expression of *EPAS1*, a zoomed-in view is included to better visualize the expression of *HIF1A* and *HIF3A*. Each data point represents a tumor or adjacent normal tissue sample collected from the same three individuals. Data are presented as mean ± SD (*n* = 3). *Padjust  < .05; **Padjust  < .01; ***Padjust  < .001. (d, e) A representative Western blot image showing HIF-2α and HIF-1α protein expression after treatment with 100 µM propranolol is shown in (d), with quantification of protein levels in (e). Each data point represents a technical replicate. Data are presented as mean ± SD of technical replicates. Statistical analysis was performed using the average value of each independent experimental run (*n* = 3), analyzed by paired t-tests. ns, not significant. Original blots are presented in S3 Fig in S1 File.

Primary HemECs were successfully isolated, and representative images of cell morphology are shown (Fig 1a). Immunofluorescence staining showed positive signals for vWF, CD31, and GLUT1 in the cultured cells (Fig 1b), consistent with a HemEC phenotype. These results are consistent with our previous study [12], which showed similar marker expression patterns in HemECs isolated using a slightly different protocol.

### HIF-related expression in IH

Bulk RNA-seq of three previously collected IH tissues and matched adjacent normal tissues [15] revealed that *EPAS1*, *HIF1A*, and hypoxia-inducible factor-3α (*HIF3A*) were all significantly upregulated in IH. *EPAS1* showed higher expression levels than *HIF1A* and *HIF3A* in these samples (Fig 1c).

A representative Western blot image of HIF-2α and HIF-1α protein expression after treatment with 100 µM propranolol is shown (Fig 1d), and the corresponding scatter plot is presented (Fig 1e). Treatment with 100 µM propranolol showed a downward trend in HIF-2α protein levels and an upward trend in HIF-1α protein levels; however, neither change reached statistical significance.

### Effects of PT-2399 on HemEC function under hypoxic conditions

**Fig 2:**
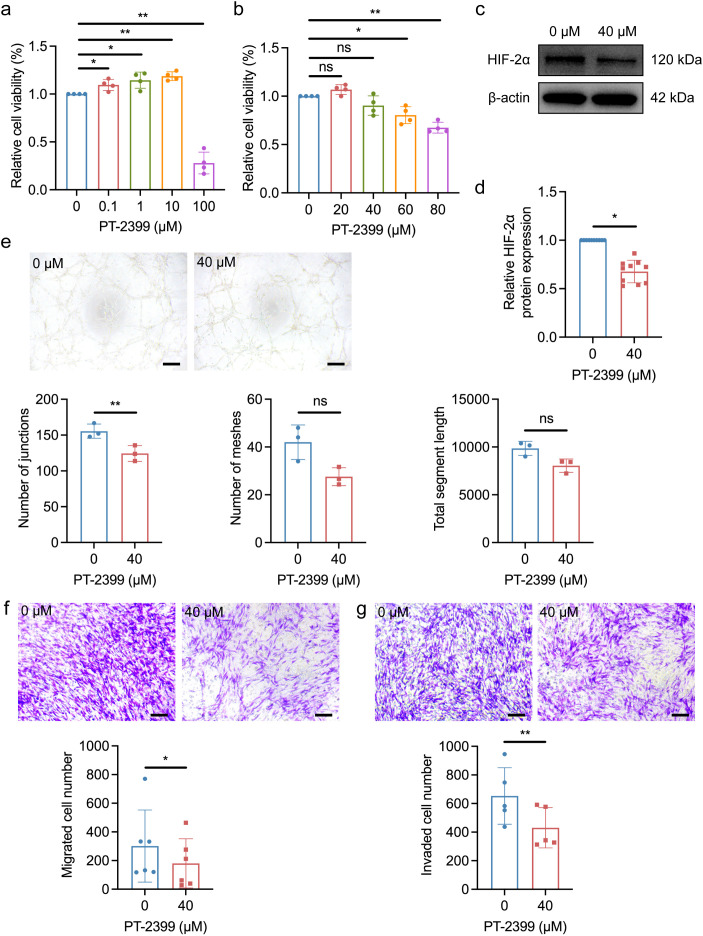
Effects of PT-2399 on HemECs function under hypoxic conditions. (a, b) CCK-8 assays were performed to assess the effects of increasing concentrations of PT-2399 on HemEC viability under hypoxic conditions. Each data point represents an independent experimental run. Data are presented as mean ± SD (*n* = 4). Statistical analysis was performed using paired t-tests. *P  <  .05; **P  <  .01; ns, not significant. (c, d) A representative Western blot image showing HIF-2α protein expression after treatment with 40 µM PT-2399 is shown in (c), with quantification of HIF-2α levels in (d). Each data point represents a technical replicate. Data are presented as mean ± SD of technical replicates. Statistical analysis was performed using the average value of each independent experimental run (*n* = 3), analyzed by a paired t-test. *P  <  .05. Original blots are presented in S4 Fig in S1 File. (e) Tube formation assays were performed under hypoxic conditions following treatment with 40 µM PT-2399. Representative images of tube formation and the quantification of the number of junctions, meshes, and total segment length are shown. Each data point represents an independent experimental run. Data are presented as mean ± SD (*n* = 3). Statistical analysis was performed using paired t-tests. **P < .01; ns, not significant. Scale bars = 500 μm. (f, g) Transwell migration and invasion assays were performed under hypoxic conditions following treatment with 40 µM PT-2399. Representative images of the migration and invasion assays and the quantification of migrated and invaded cell numbers are shown in (f) and (g). Each data point represents an independent experimental run. Data are presented as mean ± SD (migration: *n* = 6; invasion: *n* = 5). Statistical analysis was performed using paired t-tests. *P < .05; **P < .01. Scale bars = 200 μm.

To assess the effects of pharmacological HIF-2α inhibition, HemECs were treated with the selective HIF-2α inhibitor PT-2399 [13,14]. CCK-8 assays were performed to assess the effects of increasing concentrations of PT-2399 on the viability of HemECs under hypoxic conditions (Fig 2a and 2b). Under hypoxic conditions, 40 µM PT-2399 was the lowest concentration at which a downward trend in cell viability was observed without reaching statistical significance, and this concentration was selected for subsequent experiments to reduce the likelihood of nonspecific cytotoxic effects. A representative Western blot image of HIF-2α protein levels following treatment with 40 µM PT-2399 is shown (Fig 2c), and the corresponding scatter plot is presented (Fig 2d).

Tube formation assays were performed to evaluate the effects of 40 µM PT-2399 on HemECs under hypoxic conditions. Representative images and quantitative analyses of the number of junctions, meshes, and total segment length are shown (Fig 2e). The number of junctions was significantly reduced following PT-2399 treatment, whereas downward trends were observed in the number of meshes and total segment length without reaching statistical significance. Transwell migration and invasion assays were conducted to assess the motility of HemECs under hypoxia. Representative images and corresponding scatter plots show a significant reduction in cell migration (Fig 2f) and invasion (Fig 2g) following PT-2399 treatment.

### Generation and validation of *EPAS1* knockdown HemECs

**Fig 3:**
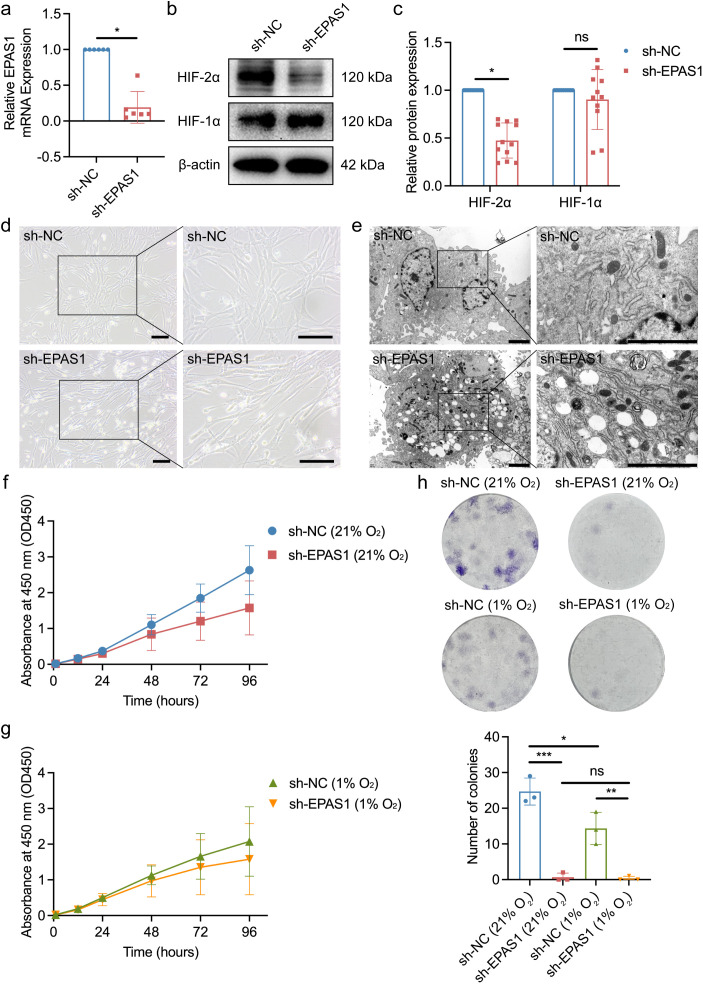
Validation of *EPAS1* knockdown and associated changes in HemEC viability and clonogenic growth. (a) *EPAS1* knockdown efficiency was assessed by qRT-PCR. Each data point represents a technical replicate. Data are presented as mean ± SD of technical replicates. Statistical analysis was performed using the average value of each independent experimental run (*n* = 4), analyzed by a paired t-test. *P < .05. (b, c) A representative Western blot image showing HIF-2α and HIF-1α protein expression after *EPAS1* knockdown is presented in (b), with quantification shown in (c). Each data point represents a technical replicate. Data are presented as mean ± SD of technical replicates. Statistical analysis was performed using the average value of each independent experimental run (*n* = 3), analyzed by paired t-tests. *P < .05; ns, not significant. Original blots are presented in S5 Fig in S1 File. (d, e) Representative light microscopy (d) and TEM (e) images showing vacuolar-like structures in *EPAS1* knockdown HemECs. Scale bars = 200 μm (d); 3 μm (e). Original TEM images are presented in S10 Fig in S1 File. (f, g) CCK-8 assays were performed following *EPAS1* knockdown under normoxic (f) and hypoxic (g) conditions. Data are presented as mean ± SD (*n* = 4). Statistical analysis was performed using repeated-measures two-way ANOVA with Geisser-Greenhouse correction. (h) Colony formation assays were performed following *EPAS1* knockdown under both conditions. Representative images of colony formation and the quantification are presented. Each data point represents an independent experimental run. Data are presented as mean ± SD (*n* = 3). Statistical analysis was performed using paired t-tests. *P < .05; **P < .01; ***P < .001; ns, not significant.

*EPAS1* knockdown was validated at the mRNA level by qRT-PCR, revealing a significant reduction in *EPAS1* expression in the sh-EPAS1 group compared to the sh-NC group (Fig 3a). A representative Western blot image of HIF-2α and HIF-1α protein expression after *EPAS1* knockdown is shown (Fig 3b), and the corresponding scatter plot is presented (Fig 3c). HIF-2α protein levels were significantly decreased in the sh-EPAS1 group, whereas HIF-1α protein levels did not differ significantly between groups. Additional Western blot images under normoxic conditions (S12 Fig in S1 File) and original blots (S13 Fig in S1 File) are shown. HIF-2α protein levels in the sh-EPAS1 group showed a downward trend compared to the sh-NC group, without reaching statistical significance.

Vacuolar-like structures were observed in the sh-EPAS1 group by light and electron microscopy. Light microscopy revealed intracellular vacuoles of varying sizes (Fig 3d), and transmission electron microscopy (TEM) showed membrane-bound vacuolar structures (Fig 3e) [16].

### Functional changes of HemECs following *EPAS1* knockdown under both normoxia and hypoxia

**Fig 4:**
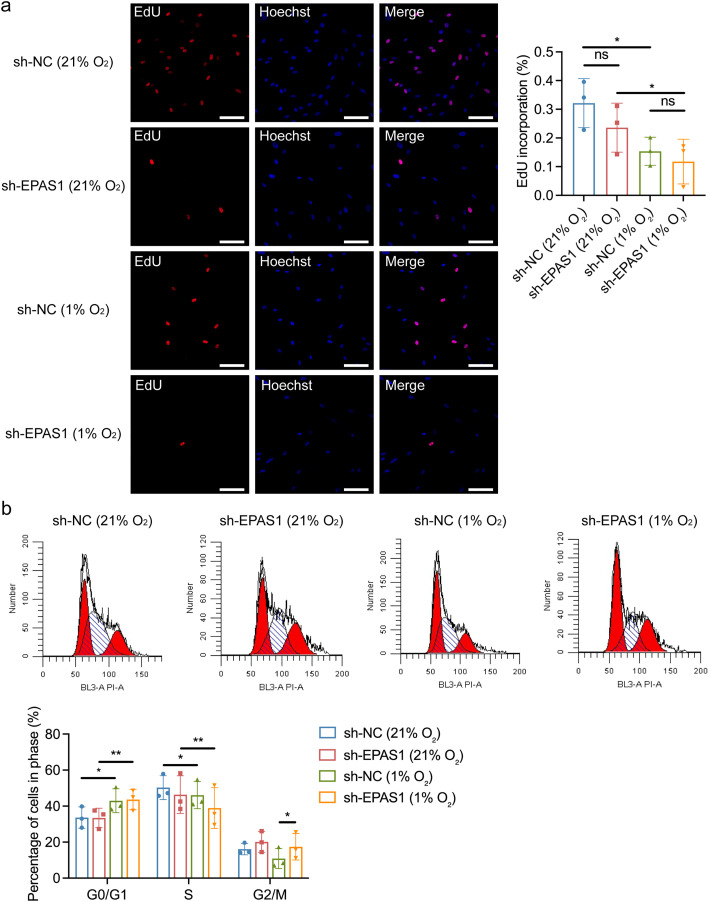
*EPAS1* knockdown–associated changes in S-phase and G2/M-phase populations in HemECs under normoxic and hypoxic conditions. (a) EdU assays were performed following *EPAS1* knockdown under both conditions. Representative images of EdU incorporation and the quantification of S-phase cell proportions are presented. Each data point represents an independent experimental run. Data are presented as mean ± SD (*n* = 3). Statistical analysis was performed using paired t-tests. *P < .05; ns, not significant. Scale bars = 100 μm. Original EdU staining images are presented in S11 Fig in S1 File. (b) Flow cytometric analysis was performed following *EPAS1* knockdown under both conditions. Representative histograms and the quantification of cell proportions in G0/G1, S, and G2/M phases are presented. Each data point represents an independent experimental run. Data are presented as mean ± SD (*n* = 3). Statistical analysis was performed using paired t-tests. *P < .05; **P < .01. Non-significant comparisons are not labeled for clarity.

CCK-8 assays (Fig 3f and 3g) showed that *EPAS1* knockdown reduced cell viability under normoxic conditions, with a significant time × knockdown status interaction (*P* = 0.0075), whereas a downward trend was also observed under hypoxic conditions, although this interaction did not reach statistical significance. Colony formation assays (Fig 3h) further showed that *EPAS1* knockdown reduced clonogenic growth under both conditions. EdU assays (Fig 4a), which were used to assess cell proliferation by detecting DNA synthesis during the S phase of the cell cycle, revealed a reduction in the proportion of S-phase cells following *EPAS1* knockdown under both normoxia and hypoxia, although the differences were not statistically significant. Flow cytometric analysis (Fig 4b) showed an increased proportion of cells in the G2/M phase under both conditions, with a significant increase observed under hypoxia. Together, *EPAS1* knockdown was associated with changes in cell cycle distribution, characterized by changes in S-phase and G2/M-phase populations.

Tube formation assays (S1a Fig in S1 File) showed increased tube formation following *EPAS1* knockdown under both normoxic and hypoxic conditions. Transwell migration assays (S1b Fig in S1 File) showed increased cell migration under both conditions, whereas invasion assays (S1c Fig in S1 File) showed a similar trend that did not reach statistical significance under hypoxia.

Overall, the patterns of functional changes observed following *EPAS1* knockdown were similar under normoxic and hypoxic conditions.

### Effects of hypoxia on HemECs

To examine the functional effects of hypoxic stimulation on HemECs, we compared cellular behaviors under normoxic and hypoxic conditions in both the sh-NC and sh-EPAS1 groups. Hypoxic stimulation was associated with reduced proliferative activity in both groups compared with normoxic conditions, as assessed by colony formation assays (Fig 3h), EdU assays (Fig 4a), and flow cytometric analysis (Fig 4b).

In colony formation assays, hypoxia was associated with a significant reduction in clonogenic growth in the sh-NC group, with a downward trend in the sh-EPAS1 group that did not reach statistical significance (Fig 3h). EdU assays revealed a reduced proportion of S-phase cells under hypoxic conditions in both groups (Fig 4a). Consistently, flow cytometric analysis showed shifts in cell cycle distribution under hypoxia within each group, characterized by an increased G0/G1-phase population and a decreased S-phase population, with a reduction in the G2/M-phase population that did not reach statistical significance (Fig 4b).

Migration and invasion assays showed that hypoxic conditions were associated with reduced migratory and invasive capacities of HemECs in both groups (S1b and S1c Fig in S1 File). Tube formation capacity did not differ significantly between normoxic and hypoxic conditions in either group (S1a Fig in S1 File).

### Bulk RNA-seq analysis of HemECs following *EPAS1* knockdown

**Fig 5:**
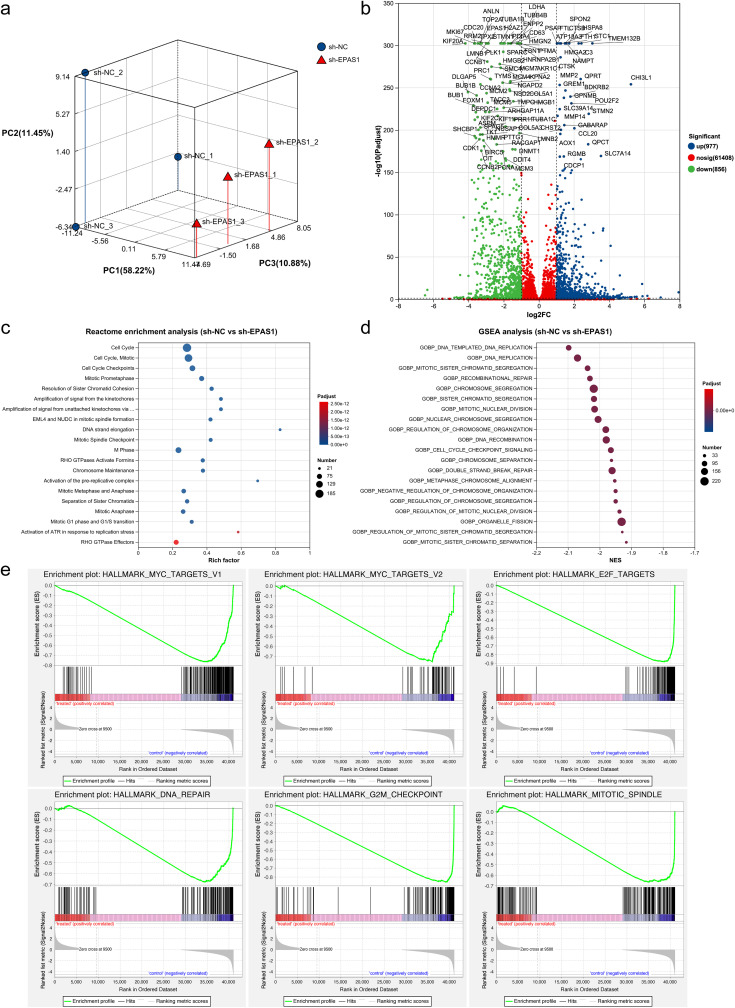
Bulk RNA-seq analysis following *EPAS1* knockdown highlights enrichment of cell cycle- and DNA replication-related programs in HemECs. (a) PCA plot of samples from the sh-NC and sh-EPAS1 groups. (b) Volcano plot showing DEGs between the sh-NC and sh-EPAS1 groups. DEGs were defined as *Padjust* < 0.05 and |log₂(fold change)| ≥ 1. The top 100 DEGs ranked by *Padjust* are labeled with gene names. (c) Reactome enrichment analysis of DEGs. The top 20 enriched pathways, ranked by *Padjust*, are displayed. (d) GSEA of all genes using the GO BP gene sets. The top 20 enriched gene sets, ranked by *Padjust*, are presented. (e) GSEA of all genes using the Hallmark gene sets. Enrichment plots of six representative gene sets are displayed.

Bulk RNA-seq analysis was performed to characterize transcriptional changes associated with *EPAS1* knockdown in HemECs. Principal component analysis (PCA) showed tight within-group clustering of samples along principal component 1 (PC1, 58.22%) (Fig 5a). Differentially expressed genes (DEGs) between the sh-NC and sh-EPAS1 groups were visualized using a volcano plot, identifying 977 upregulated and 856 downregulated genes in the sh-EPAS1 group (Fig 5b).

Reactome enrichment analysis of DEGs revealed significant enrichment in pathways related to the cell cycle and DNA replication (Fig 5c). Consistently, gene set enrichment analysis (GSEA) of all genes using GO Biological Process (GO BP) gene sets showed enrichment of gene sets associated with the cell cycle and DNA replication (Fig 5d). GSEA of all genes using the Hallmark gene sets further identified significant negative enrichment of MYC targets, E2F targets, DNA repair, G2/M checkpoint, and mitotic spindle signatures (Fig 5e; S1 Table in S1 File).

Together, these analyses showed enrichment of transcriptional programs related to the cell cycle and DNA replication in *EPAS1* knockdown HemECs.

### *EPAS1* knockdown is associated with changes in cell cycle- and DNA replication-related proteins

**Fig 6:**
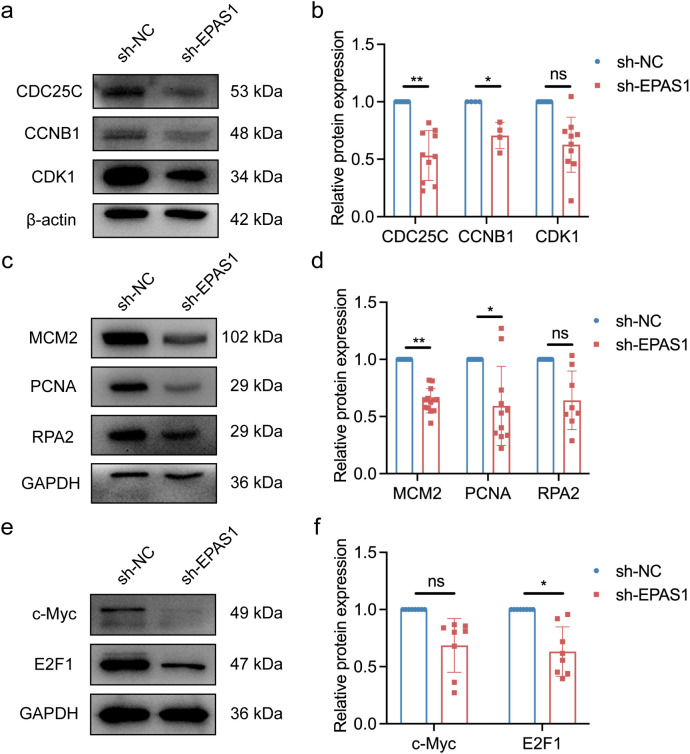
*EPAS1* knockdown-associated changes in cell cycle- and DNA replication-related proteins in HemECs. (a, b) A representative Western blot image showing CDC25C, CCNB1, and CDK1 protein expression following *EPAS1* knockdown is presented in (a), with quantification shown in (b). (c, d) A representative Western blot image showing MCM2, PCNA, and RPA2 protein expression is presented in (c), with quantification shown in (d). (e, f) A representative Western blot image of c-Myc and E2F1 is shown in (e), with quantification shown in (f). Each data point represents a technical replicate. Data are presented as mean ± SD of technical replicates. Statistical analysis was performed using the average value of each independent experimental run (*n* = 3), analyzed by paired t-tests. *P < .05; **P < .01; ns, not significant. Original blots corresponding to panels (a), (c), and (e) are presented in S6, S7, and S8 Figs in S1 File, respectively.

Protein expression levels of selected G2/M-related proteins were examined by Western blotting. A representative image of cell division cycle 25C (CDC25C), cyclin B1 (CCNB1), and cyclin-dependent kinase 1 (CDK1) is shown (Fig 6a), and the corresponding scatter plot is presented (Fig 6b). *EPAS1* knockdown was associated with a significant reduction in CDC25C and CCNB1 protein levels, with CDK1 exhibiting a decreasing trend that did not reach statistical significance.

To assess proteins involved in DNA replication, protein expression levels of minichromosome maintenance complex component 2 (MCM2), proliferating cell nuclear antigen (PCNA), and replication protein A2 (RPA2) were analyzed. A representative Western blot image is shown (Fig 6c), and the corresponding scatter plot is presented (Fig 6d). *EPAS1* knockdown was associated with a significant reduction in MCM2 and PCNA protein levels, with RPA2 showing a downward trend without reaching statistical significance.

Based on the MYC- and E2F-related transcriptional signatures observed in RNA-seq analyses, the protein levels of cellular myelocytomatosis oncogene (c-Myc) and E2F transcription factor 1 (E2F1) were examined. A representative Western blot image is shown (Fig 6e), and the corresponding scatter plot is presented (Fig 6f). *EPAS1* knockdown was associated with a significant reduction in E2F1 protein levels, accompanied by a non-significant decrease in c-Myc protein levels.

## Discussion

This study has several important limitations that should be considered when interpreting the findings.

First, all HemEC-based cellular experiments (functional assays, transcriptomic profiling, and protein analyses) were performed using cells derived from a single IH specimen. Accordingly, the present findings do not capture inter-patient heterogeneity and should be regarded as exploratory observations from independent experimental runs derived from the same source rather than patient-level biological replication. Although this constraint limits the generalizability of our data, the relevance of HIF-2α to IH is supported by multiple independent observations, including immunohistochemical evidence of HIF-2α positivity in IH tissues [8], transcriptomic evidence of differential *EPAS1* expression across IH disease phases and in response to propranolol [9], and significant *EPAS1* upregulation in our earlier bulk RNA-seq analysis of IH tissues compared with matched adjacent normal tissues [15]. Together, these observations support the rationale for examining HIF-2α in IH while underscoring the need for future validation in additional patient-derived systems.

Second, HIF-2α perturbation was achieved using a single shRNA construct for *EPAS1* knockdown and a pharmacological inhibitor, without the inclusion of rescue experiments or validation using multiple independent genetic constructs. Although *EPAS1* knockdown efficiency was confirmed at both mRNA and protein levels, off-target effects cannot be excluded. As such, the present study does not establish definitive HIF-2α-dependent causal relationships but rather delineates HIF-2α-associated cellular and transcriptional alterations.

Within these limitations, the present study provides several consistent observations regarding HIF-2α in HemECs. Across multiple experimental approaches, *EPAS1* knockdown was associated with changes in HemEC proliferation and cell cycle distribution, together with enrichment of DNA replication/cell cycle-related transcriptional programs. These cellular effects were observed under both normoxic and hypoxic conditions. Notably, HIF-2α-associated phenotypic trends were similar under normoxic and hypoxic conditions.

An important consideration in interpreting the present findings is the discrepancy observed between pharmacological inhibition of HIF-2α using PT-2399 and shRNA-mediated *EPAS1* knockdown. In hypoxic HemEC cultures, PT-2399 treatment was associated with reduced migratory and invasive capacities and with a significant reduction in junction number in tube formation assays, with downward trends in mesh number and total segment length, whereas cell viability at the selected dose showed a downward trend without reaching statistical significance. By contrast, *EPAS1* knockdown was associated with a more complex phenotype characterized by impaired proliferation and altered cell cycle distribution, alongside increased tube formation and migration under both oxygen conditions and increased invasion under normoxia (with a similar directional trend under hypoxia), and was accompanied by cell cycle/DNA replication-related molecular changes. These divergent outcomes indicate that pharmacological and genetic perturbation of HIF-2α are not functionally equivalent in this cellular context and should not be interpreted as mutually reinforcing evidence of a single HIF-2α-dependent effect.

Several non-mutually exclusive factors may contribute to this non-concordance. Pharmacological inhibition and shRNA-mediated *EPAS1* knockdown perturb HIF-2α through distinct mechanisms and time scales—PT-2399 disrupts HIF-2α transcriptional activity by inhibiting HIF-2α/HIF-1β heterodimerization [13,14], whereas shRNA-mediated knockdown produces sustained reduction of *EPAS1* expression—and therefore need not yield identical cellular outcomes. In addition, compound potency and functional specificity can be cell type and context dependent, whereas long-term genetic knockdown may elicit adaptive or secondary cellular responses over time; both approaches are also subject to potential off-target effects.

Because we did not perform additional on-target validation in HemECs, such as rescue experiments, multiple independent knockdown constructs, or testing an additional HIF-2α inhibitor with distinct chemistry, the specificity of either perturbation cannot be established definitively. Accordingly, the PT-2399 results should be interpreted as exploratory functional observations under hypoxic culture conditions. For the same reason, the *EPAS1* knockdown findings are interpreted here as phenomena associated with *EPAS1* knockdown rather than definitive evidence of HIF-2α-dependent function. Although the knockdown data show associations across functional assays, transcriptomic signatures of cell cycle/DNA replication programs, and directionally consistent protein-level changes, further validation will be needed to confirm which phenotypes are strictly HIF-2α-dependent.

*EPAS1* knockdown also produced a dissociated pattern of functional changes across assays. While proliferative capacity was impaired and cell cycle distribution was altered, tube formation and migration increased under both normoxic and hypoxic conditions, and invasion increased under normoxia (with a similar directional trend under hypoxia). These directionally distinct phenotypes indicate that the effects associated with *EPAS1* knockdown did not follow a single uniform direction across assays.

In addition, *EPAS1* knockdown was accompanied by vacuolar-like structures on light and electron microscopy and by changes in transcript levels of microtubule-associated protein 1 light chain 3 beta (*MAP1LC3B*), sequestosome 1 (*SQSTM1*), and synaptosome associated protein 29 (*SNAP29*) in RNA-seq data (S2a Fig in S1 File) [16]. These findings are descriptive and should be interpreted as associated observations rather than evidence of a defined autophagy pathway or altered autophagic flux.

Transcriptomic analyses provide potential clues to these motility-associated changes. *EPAS1* knockdown was accompanied by downregulation of tissue inhibitor of metalloproteinases 1 (*TIMP1*) mRNA and upregulation of multiple matrix metalloproteinase (*MMP*) mRNAs (S2b Fig in S1 File), a pattern that could facilitate extracellular matrix remodeling and is consistent with altered migratory/invasive behavior [17]. Markers commonly used to indicate pro-angiogenic activation were not broadly or consistently upregulated, with vascular endothelial growth factor A (*VEGFA*) as a partial exception (S2c Fig in S1 File). This is compatible with the possibility that increased tube formation metrics after *EPAS1* knockdown arise indirectly through altered motility and matrix remodeling rather than through broad activation of canonical angiogenic gene-expression programs. Given that the HIF-2α–TIMP1 relationship has been reported inconsistently across contexts [18,19], and rescue experiments were not performed, these observations should be interpreted as associative and hypothesis-generating.

Taken together, *EPAS1* knockdown was most consistently associated with reduced proliferative capacity and cell cycle/DNA replication-related programs, while motility- and tube formation-related changes were directionally distinct across assays.

Hypoxia has long been proposed as a contributor to IH pathogenesis. To assess the functional effects of hypoxic stimulation, we focused on the sh-NC group as an approximation of untreated HemECs. Under the 1% O₂ condition used here, hypoxia was associated with reduced proliferative capacity and decreased migratory/invasive activity compared with normoxia, whereas tube formation capacity was not significantly altered. Although 1% O₂ is commonly used in hypoxia-related experimental settings, including endothelial cell and IH studies [20–22], hypoxic responses can vary with oxygen tension, exposure duration, and cellular state. Moreover, in vitro culture conditions cannot fully recapitulate the complex tumor microenvironment in vivo. Accordingly, the present in vitro results should not be interpreted as definitively excluding a role for hypoxia in IH.

In parallel, *EPAS1* knockdown produced broadly comparable phenotypic effects under both normoxic and hypoxic conditions, with no clear evidence that hypoxic stimulation enhanced the magnitude of the knockdown-associated changes. Together, these observations are compatible with the possibility that HIF-2α-related cellular regulation in HemECs in this in vitro system is not strongly dependent on exogenous hypoxic stimulation.

One potential explanation is pseudohypoxia, in which hypoxia-independent mechanisms maintain HIF-related signaling under normoxic conditions [10]. In the present context, this could plausibly involve altered oxygen-sensing regulation of HIF-2α stability. In line with this possibility, analysis of our IH tissue transcriptomic dataset [15] showed reduced expression of Egl-9 family hypoxia-inducible factor 3 (*EGLN3*), which encodes prolyl hydroxylase domain–containing protein 3 (PHD3), an oxygen-sensing prolyl hydroxylase implicated in regulating HIF-α stability, including HIF-2α [23]. Among the *EGLN* genes (*EGLN1–3*) examined in this dataset, *EGLN3* showed the most prominent decrease, suggesting the *EGLN3*/PHD3 axis as a plausible candidate for hypoxia-independent modulation of HIF-2α (S2d Fig in S1 File). While this observation does not establish causality, it provides a testable hypothesis that reduced *EGLN3* expression may contribute to sustained HIF-2α activity under normoxic conditions in IH. Notably, *EPAS1* mRNA was also increased in IH tissues, suggesting that transcriptional upregulation and reduced oxygen-sensing-dependent degradation are not mutually exclusive and may together shape the elevated HIF-2α state observed in IH.

Our HemEC datasets support an association between *EPAS1* knockdown and proliferative programs related to the cell cycle and DNA replication. To reduce confounding from hypoxia-driven global transcriptional remodeling, transcriptomic profiling and protein analyses were performed under normoxic conditions. In this setting, *EPAS1* knockdown was associated with negative enrichment of MYC- and E2F-related gene sets and with directionally consistent downward shifts in c-Myc/E2F1 and multiple regulators of G2/M progression and DNA replication (with some changes reaching statistical significance and others showing directionally consistent trends). Overall, these molecular patterns were broadly aligned with the observed functional phenotypes (reduced clonogenic growth and a shift toward G2/M accumulation). Consistent with prior work showing that HIF-2α can enhance c-Myc transcriptional activity [24,25], our findings are compatible with an HIF-2α–c-Myc/E2F connection in proliferative regulation. Further work will be needed to establish how directly these associations depend on HIF-2α.

In summary, this study highlights HIF-2α as an underexplored factor in HemEC biology. *EPAS1* knockdown was most consistently associated with proliferative programs related to the cell cycle and DNA replication. The observation that *EPAS1* knockdown-associated phenotypic trends were similar under normoxic and hypoxic conditions is compatible with the possibility of a hypoxia-independent component of HIF-2α regulation in this system. Future work using additional patient-derived models and stronger on-target validation will be needed to define causal relationships and to clarify how the dissociated motility- and tube formation-related phenotypes relate to the proliferation- and cell cycle-associated changes observed here.

## Materials and methods

### Isolation, culture, and identification of HemECs

A proliferating IH specimen was obtained from the chest and abdominal region of a 5-month-old male infant undergoing surgical resection at the Department of Plastic and Aesthetic Surgery, Shandong Provincial Hospital under approved protocols (see Ethics statement).

Following tissue collection, primary HemECs were isolated. The IH sample was digested with dispase II (Sigma-Aldrich, D4693) and a mixture of collagenase from *Clostridium histolyticum* (Sigma-Aldrich, C0130) and DNase I (Roche, 10104159001). The isolated HemECs were cultured in complete Endothelial Cell Medium (ECM; ScienCell, 1001), supplemented with 5% fetal bovine serum (FBS), 1% endothelial cell growth supplement (ECGS), and 1% penicillin-streptomycin (P/S). The cells were cultured and expanded to obtain a high-purity population, leveraging the proliferative advantage of HemECs. To assess the purity of the cultured population, immunofluorescence staining was performed using HemECs-specific markers, including von Willebrand factor (vWF), cluster of differentiation 31 (CD31), and glucose transporter 1 (GLUT1).

### Immunofluorescence staining

Cells were seeded in 24-well plates and cultured for 24 h. They were then fixed with 4% paraformaldehyde (PFA), permeabilized with 0.3% Triton X-100, and blocked with goat serum. Primary antibodies against vWF (Diagbio, db15183), CD31 (Proteintech, 66065–2-Ig), and GLUT1 (Diagbio, db15983) were applied, followed by fluorophore-conjugated secondary antibodies (Proteintech, RGAR004 and RGAM002). Nuclei were counterstained with DAPI. Images were acquired using a Zeiss LSM 900 confocal microscope equipped with an Airyscan detector and a 20 × Plan-type objective lens. DAPI channel was pseudo-coloured in blue for presentation.

### Bulk RNA-seq analysis of IH tissues

RNA-seq data used in this study were derived from our previously published work [15], in which bulk RNA-seq was performed on IH tissues and matched adjacent normal tissues (*n* = 3 biological replicates per group) under approved institutional protocols (see Ethics statement). Raw sequencing reads were normalized as fragments per kilobase of transcript per million mapped reads (FPKM). Differential expression analysis was performed using DESeq2 based on raw read count data, and *padjust* were reported. Although the expression of specific genes analyzed here was not included in the original publication, the *padjust* and FPKM values were retained and used for visualization in the present study.

### Western blotting

Unless otherwise specified, all cells were cultured under normoxic conditions and total protein was extracted without hypoxic exposure. For Western blotting detection of HIF-2α and, where applicable, HIF-1α in the main validation experiments (Figs 2c, 2d, 3b, and 3c), cells were exposed to hypoxic conditions (1% O₂) for 8 h immediately prior to protein extraction to allow protein stabilization and ensure reliable detection. Additional Western blotting detection of HIF-2α following *EPAS1* knockdown under normoxic conditions (21% O₂) was also performed (S12 Fig in S1 File).

Total protein was extracted using RIPA buffer containing 1% protease and phosphatase inhibitors. Protein concentrations were determined and adjusted using a BCA assay. Equal amounts of protein were separated by 10% SDS-PAGE and transferred to PVDF membranes. After transfer, membranes were cut into sections according to molecular weight ranges prior to incubation with the respective primary antibodies. For proteins with similar molecular weights that could not be separated by membrane cutting, membranes were sequentially reprobed after stripping with a mild stripping buffer. The membranes were incubated overnight at 4 °C with primary antibodies against HIF-2α (Proteintech, 26422–1-AP), HIF-1α (Proteintech, 66730–1-Ig), β-actin (Proteintech, 66009–1-Ig), CDC25C (Diagbio, db15720), CCNB1 (Diagbio, db13216), CDK1 (Diagbio, db12527), MCM2 (Diagbio, db11270), PCNA (Diagbio, db11523), RPA2 (Diagbio, db11166), GAPDH (Diagbio, db11729), c-Myc (Diagbio, db14926), and E2F1 (Diagbio, db12146). The membranes were incubated with HRP-conjugated goat anti-rabbit IgG (H + L) and goat anti-mouse IgG (H + L) secondary antibodies (ABclonal, AS014 and AS003) at room temperature for 1 h, followed by signal development using an enhanced chemiluminescence (ECL) detection kit. Band intensities were quantified using ImageJ and normalized to matched controls within each replicate.

### Cell culture under normoxic and hypoxic conditions

Cells were cultured under normoxic (21% O₂, 5% CO₂) or hypoxic (1% O₂, 5% CO₂, 94% N₂) conditions in CO₂ incubators (Thermo Fisher), with hypoxia maintained using a ProOx C21 O₂ controller (BioSpherix).

### Quantitative real-time PCR (qRT-PCR)

All cells were cultured under normoxic conditions and total RNA was extracted without hypoxic exposure.

Total RNA was extracted using a standard column-based RNA isolation kit. cDNA was synthesized from 1 μg of total RNA using a reverse transcription kit with genomic DNA removal. qPCR was performed using SYBR Green master mix on a real-time PCR system. Amplification and melting curves were analyzed to assess the specificity of the PCR products. Relative mRNA levels were calculated using the 2^ − ΔΔCT method, with *ACTB* as the reference gene. The primers used are listed in S2 Table in S1 File.

### CCK-8 assay after PT-2399 treatment

HemECs were seeded in 96-well plates and treated with PT-2399 (Proteintech, 1672662-14-4), which was diluted in complete ECM to the indicated concentration. Cells were then subjected to hypoxic treatment (1% O₂) for 48 h. After treatment, the medium was replaced with 110 μL of fresh complete ECM containing 10 μL of CCK-8 per well. After 3 h of incubation, absorbance at 450 nm was measured using a microplate reader and normalized to the 0 µM control within each replicate.

### Tube formation assay after PT-2399 treatment

50 μL of Matrigel (Mogengel Biotech, 082704) was added to 96-well plates. HemECs were suspended in 100 μL of complete ECM and seeded onto the Matrigel-coated wells. PT-2399 was added to the 150 μL total volume at a concentration of 40 μM. Cells were then subjected to hypoxic treatment (1% O₂). Tube formation was monitored every 2 h. Images were captured at the time point when at least one group showed mature tube-like structures. Quantification of tube formation was performed using the Angiogenesis Analyzer plugin in ImageJ.

### Transwell migration assay after PT-2399 treatment

HemECs were seeded in the upper chambers of tissue culture inserts (Biofil, TCS003024) in ECM, and complete ECM was added to the lower chambers. PT-2399 was added to both chambers at a concentration of 40 μM. Cells were then subjected to hypoxic treatment (1% O₂) for 48 h. Cells on the lower membrane were fixed with 4% PFA and stained with 0.1% crystal violet solution.

### Transwell invasion assay after PT-2399 treatment

A mixture of 2.5 μL Matrigel and 97.5 μL ECM was added to the upper chambers of the inserts. After removing unbound components, the assay was performed as described for the transwell migration assay after PT-2399 treatment.

### Lentiviral-mediated gene knockdown of *EPAS1*

*EPAS1* knockdown lentiviruses were constructed using the GV493 vector by Shanghai GeneChem (China), with the target sequence CAGTACCCAGACGGATTTCAA for sh-EPAS1. The negative control lentivirus (sh-NC) contained a non-targeting shRNA sequence (TTCTCCGAACGTGTCACGT). HemECs were infected with sh-NC or sh-EPAS1 lentivirus, followed by puromycin selection.

### Transmission Electron Microscopy (TEM)

Cells were fixed with 2.5% glutaraldehyde followed by 1% osmium tetroxide. After standard dehydration, embedding, ultrathin sectioning (60–90 nm), and staining with uranyl acetate and lead citrate, samples were imaged using a TEM (JEM-1400FLASH, JEOL). Each copper grid was initially examined at 6000 × magnification to survey the overall structure, and representative regions were subsequently imaged at 20,000 × magnification. TEM sample processing and imaging were performed by Lilai Biotechnology (China).

### CCK-8 assay after *EPAS1* knockdown

HemECs infected with sh-NC or sh-EPAS1 lentivirus were seeded in 96-well plates. After 2 hours of incubation, the medium was replaced with 110 μL of fresh complete ECM containing 10 μL of CCK-8 per well. Absorbance at 450 nm was measured at 1, 12, 24, 48, 72, and 96 h after CCK-8 addition. After the 1-hour measurement, cells were incubated under normoxic (21% O₂) or hypoxic (1% O₂) conditions according to the experimental groups.

### Colony formation assay

HemECs infected with sh-NC or sh-EPAS1 lentivirus were seeded at a density of 1000 cells/well in 6-well plates and incubated for 48 h. Cells were then incubated under normoxic (21% O₂) or hypoxic (1% O₂) conditions according to the experimental groups. The colonies were fixed with 4% PFA and stained with 0.1% crystal violet solution when at least one group of cells formed colonies sufficiently. Images were captured, and the number of visible colonies was counted.

### EdU proliferation assay

HemECs infected with sh-NC or sh-EPAS1 lentivirus were seeded in 35 mm glass bottom dishes and incubated for 24 h. Cells were then incubated under normoxic (21% O₂) or hypoxic (1% O₂) conditions for 24 h according to the experimental groups. Subsequently, cells were incubated with EdU for 2 h and processed using the EdU Imaging Kit (Cy5) (APExBIO, K1076) following the manufacturer’s protocol. Images were acquired using a Zeiss LSM 900 confocal microscope equipped with an Airyscan detector and a 20 × Plan-type objective lens. Hoechst channel was pseudo-coloured in blue for presentation.

### Flow cytometry analysis of the cell cycle

HemECs infected with sh-NC or sh-EPAS1 lentivirus were seeded in 6-well plates and incubated for 24 h. Cells were then incubated under normoxic (21% O₂) or hypoxic (1% O₂) conditions for 24 h according to the experimental groups. Subsequently, cells were fixed with 70% ethanol at 4 °C overnight and stained with PI/RNase Staining Solution (SIMUBIOTECH, CY001-L) following the manufacturer’s protocol. Cell cycle analysis was performed by flow cytometry, and the data were analyzed using ModFit LT 5.0.

### Tube formation assay after *EPAS1* knockdown

HemECs infected with sh-NC or sh-EPAS1 lentivirus were suspended in 100 μL of complete ECM and seeded onto the Matrigel-coated wells. Cells were incubated under normoxic (21% O₂) or hypoxic (1% O₂) conditions according to the experimental groups. The rest of the procedure was performed as described for PT-2399 treatment.

### Transwell migration assay after *EPAS1* knockdown

HemECs infected with sh-NC or sh-EPAS1 lentivirus were seeded in the upper chambers of the inserts in ECM, and complete ECM was added to the lower chambers. Cells were incubated under normoxic (21% O₂) or hypoxic (1% O₂) conditions for 24 h according to the experimental groups. The rest of the procedure was performed as described for PT-2399 treatment.

### Transwell invasion assay after *EPAS1* knockdown

A mixture of 2.5 μL Matrigel and 97.5 μL ECM was added to the upper chambers of the inserts. After removing unbound components, the assay was performed as described for the transwell migration assay after *EPAS1* knockdown.

### Statistical analysis

All statistical analyses for experiments, except for RNA-seq data, were performed using GraphPad Prism (v10.2.0). Repeated-measures two-way ANOVA with Geisser-Greenhouse correction was used to analyze CCK-8 assay results after *EPAS1* knockdown, with time (1, 12, 24, 48, 72, and 96 h) and knockdown status (sh-NC vs sh-EPAS1) as factors. Other data were analyzed using two-tailed paired t-tests, with pairing defined at the level of the independent experimental run, in which control and experimental samples were processed in parallel under matched experimental conditions. *P* < 0.05 was considered statistically significant.

### Bulk RNA-seq and transcriptomic analysis after *EPAS1* knockdown

Bulk RNA-seq was performed by Majorbio (China) for sequencing and initial analysis, with further data mining and visualization conducted by the authors on the Majorbio cloud platform. Samples were from two groups of cells cultured under normoxic conditions: sh-NC and sh-EPAS1 (*n* = 3 samples per group).

### Principal component analysis (PCA)

Raw read count data were processed using RSEM to obtain transcripts per million (TPM) values. PCA was then performed on the TPM expression matrix, and a 3D PCA plot was generated for visualization.

### Differentially expressed genes (DEGs)

Differential expression analysis was performed using DESeq2 based on raw read count data. *P*-values were adjusted for multiple testing using the Benjamini-Hochberg (BH) method. Genes with a *Padjust* < 0.05 and |log₂(fold change)| ≥ 1 were considered significantly differentially expressed. The significant DEGs were visualized in a volcano plot, with the top 100 genes ranked by *Padjust* labeled with gene names.

### Reactome enrichment analysis

Significant DEGs were imported into the Reactome database for pathway enrichment analysis. Fisher’s exact test was used to assess the enrichment of genes in each pathway. *P*-values were adjusted for multiple testing using the BH method. Pathways with *Padjust* < 0.05 were considered significantly enriched. The top 20 pathways were presented based on *Padjust*.

### Gene set enrichment analysis (GSEA)

GSEA was performed using the full TPM expression matrix to identify functionally enriched gene sets. The GO BP gene sets from the MSigDB database were used as the reference. Genes were ranked based on the signal-to-noise metric, and enrichment scores (ES) were calculated for each gene set. Normalized enrichment scores (NES) and *P*-values were then computed to evaluate significance. *Padjust*, representing the false discovery rate (FDR), was used to determine significantly enriched gene sets. The top 20 gene sets were identified based on *Padjust*. Subsequently, the Hallmark gene sets from the MSigDB database were used for further analysis, following the same procedure. The top 10 gene sets were identified based on *Padjust*, and enrichment plots were presented for selected representative gene sets.

### Ethics statement

This study was conducted in accordance with the Declaration of Helsinki. Human tissue samples used in this study were originally collected during routine surgical treatment of IH and were collected under an ethically approved protocol (NSFC: no. 2021−743) [12]. Written informed consent was obtained from the legal guardians of all participants at the time of sample collection. The tissue-derived RNA-seq data analyzed in this study were derived from our previously published work [15], in which all procedures involving human tissues had been ethically approved at the time of the original study. No new human tissue collection or tissue-derived RNA-seq was conducted for the present analysis. The current study, which involved the research use of previously collected biospecimens and previously generated transcriptomic data, was approved by the Biomedical Research Ethics Committee of Shandong Provincial Hospital (SWYX: No. 2025−323). The study data were accessed for research purposes as part of the analyses reported in this manuscript on 12 June 2025. Authors did not have access to information that could identify individual participants during or after data collection.

## Supporting information

S1 File This file contains S1 Table, S2 Table, and S1–S13 Figs. (DOCX)

S2 File This file contains the original images underlying the Western blot results. (PDF)
