# Supplementary material for: EPAS1 knockdown is associated with cell cycle and DNA replication programs and MYC/E2F-related signatures in hemangioma endothelial cells
Source: PLoS One. 2026 Jul 24;21(7):e0354272. doi: 10.1371/journal.pone.0354272 (PMC13399520; doi:10.1371/journal.pone.0354272)
Supplement: S1 File — (DOCX) [file pone.0354272.s001.docx]

**S1 Table. GSEA analysis of the top 10 Hallmark gene sets based on *Padjust* in bulk RNA-seq results.**

| Gene Set Name | Group | NES | *Padjust* |
| --- | --- | --- | --- |
| HALLMARK_E2F_TARGETS | control | -2.2264752 | 0 |
| HALLMARK_MYC_TARGETS_V1 | control | -1.9350249 | 0 |
| HALLMARK_INTERFERON_ALPHA_RESPONSE | control | -1.8089541 | 0 |
| HALLMARK_G2M_CHECKPOINT | control | -2.1762824 | 0 |
| HALLMARK_DNA_REPAIR | control | -1.6694771 | 0.000222487 |
| HALLMARK_MITOTIC_SPINDLE | control | -1.6838831 | 0.000266984 |
| HALLMARK_MYC_TARGETS_V2 | control | -1.6464435 | 0.000368254 |
| HALLMARK_OXIDATIVE_PHOSPHORYLATION | control | -1.6598839 | 0.000420862 |
| HALLMARK_SPERMATOGENESIS | control | -1.5789698 | 0.002423551 |
| HALLMARK_INTERFERON_GAMMA_RESPONSE | control | -1.5459068 | 0.003832809 |

Note: “Group” indicates the sample group in which the gene set is positively enriched.

**S2 Table. Primers used in this manuscript.**

| GENE | Forward Primer (5’-3’) | Reverse Primer (5’-3’) |
| --- | --- | --- |
| *ACTB* | CATGTACGTTGCTATCCAGGC | CTCCTTAATGTCACGCACGAT |
| *EPAS1* | TTGCTCTGAAAACGAGTCCGA | GGTCACCACGGCAATGAAAC |

**
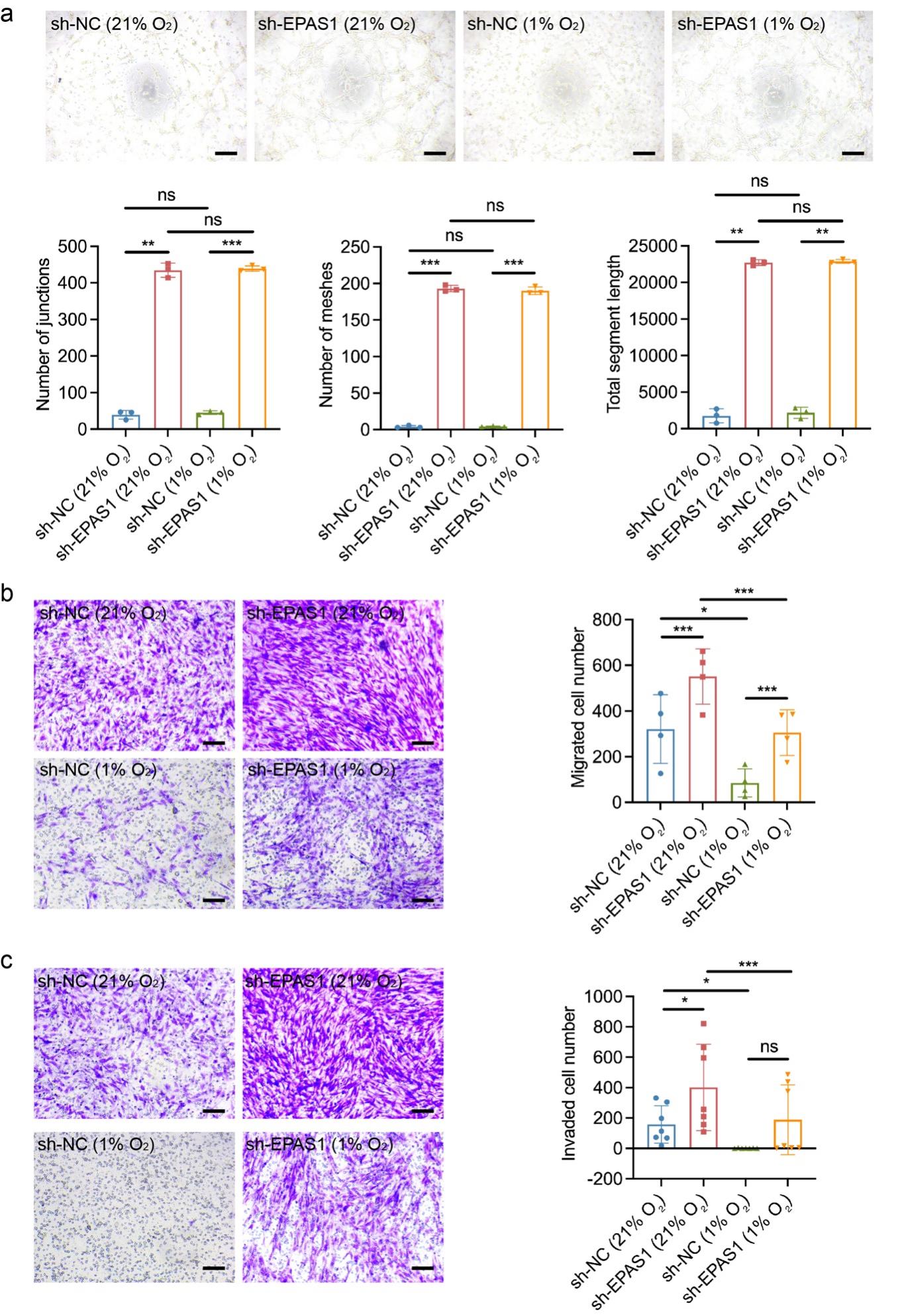
**

**S1** **Fig. *EPAS1* knockdown-associated changes in tube formation, migration, and invasion of HemECs under normoxic and hypoxic conditions.** (**a**) Tube formation assays were performed following *EPAS1* knockdown under both conditions. Representative images of tube formation and the quantification of the number of junctions, meshes, and total segment length are shown. Each data point represents an independent experimental run. Data are presented as mean ± SD (*n* = 3). Statistical analysis was performed using paired *t*-tests. ***P* < .01; ****P* < .001; ns, not significant. Scale bars = 500 μm. (**b, c**) Transwell migration and invasion assays were performed following *EPAS1* knockdown under both conditions. Representative images of the migration and invasion assays and the quantification of migrated and invaded cell numbers are shown in (b) and (c). Each data point represents an independent experimental run. Data are presented as mean ± SD (migration: *n* = 4; invasion: *n* = 7). Statistical analysis was performed using paired *t*-tests. **P* < .05; ****P* < .001; ns, not significant. Scale bars = 200 μm.

**
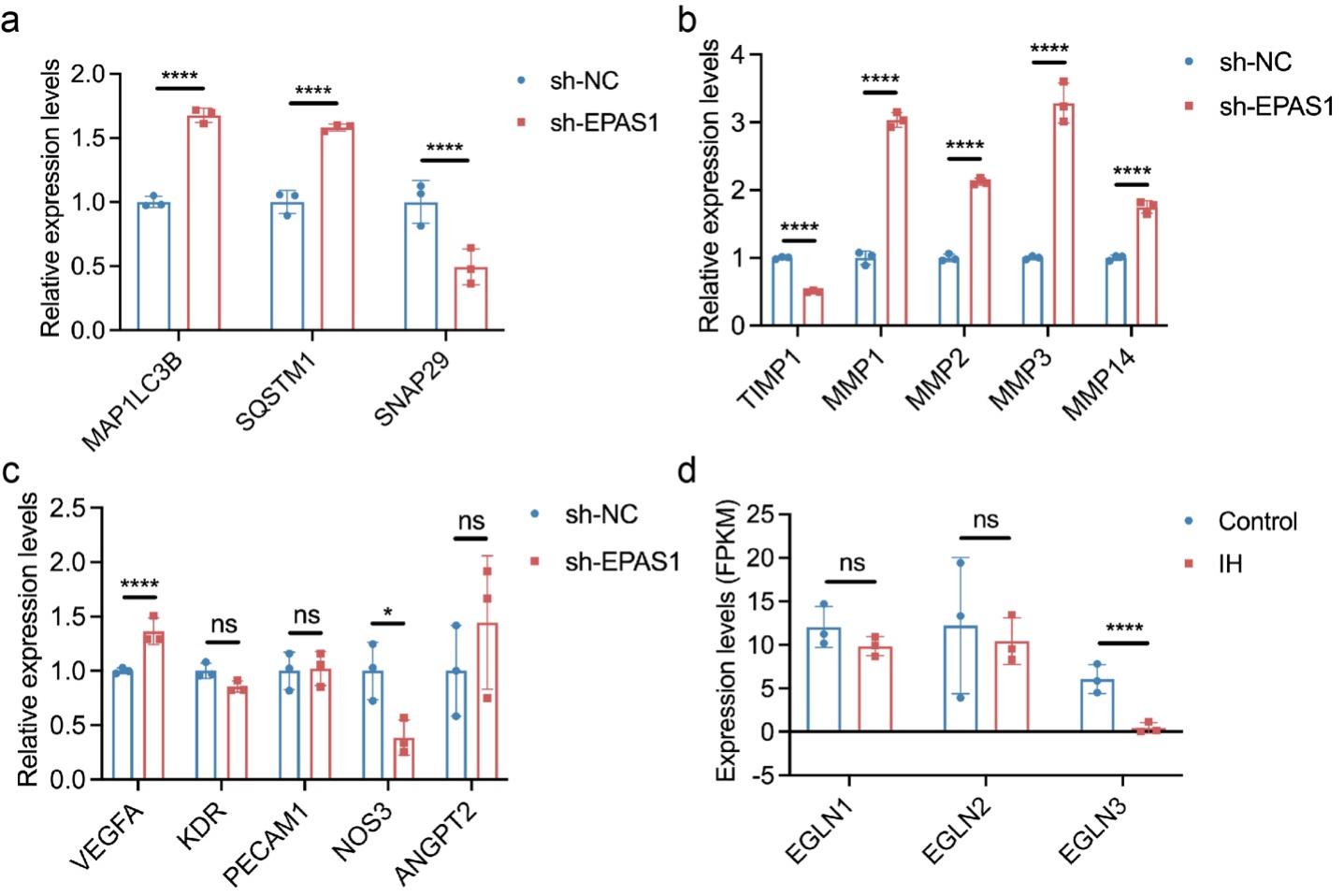
**

**S2 Fig. Bulk RNA-seq analysis of selected gene expression patterns in *EPAS1* knockdown HemECs and IH tissues.** (**a–c**) RNA-seq analysis of *EPAS1* knockdown HemECs showing mRNA expression levels of selected genes commonly used as autophagy-associated markers (a), selected motility- and matrix remodeling-associated genes (b), and selected genes commonly used as indicators of pro-angiogenic activation (c). Each data point represents a sample. Data are presented as mean ± SD (*n* = 3). Expression levels are normalized to the sh-NC group. **Padjust* < .05; *****Padjust* < .0001; ns, not significant. (**d**) RNA-seq analysis of IH tissues showing mRNA expression levels of *EGLN* family members. Each data point represents a tumor or adjacent normal tissue sample collected from the same three individuals. Data are presented as mean ± SD (*n* = 3). *****Padjust* < .0001; ns, not significant.


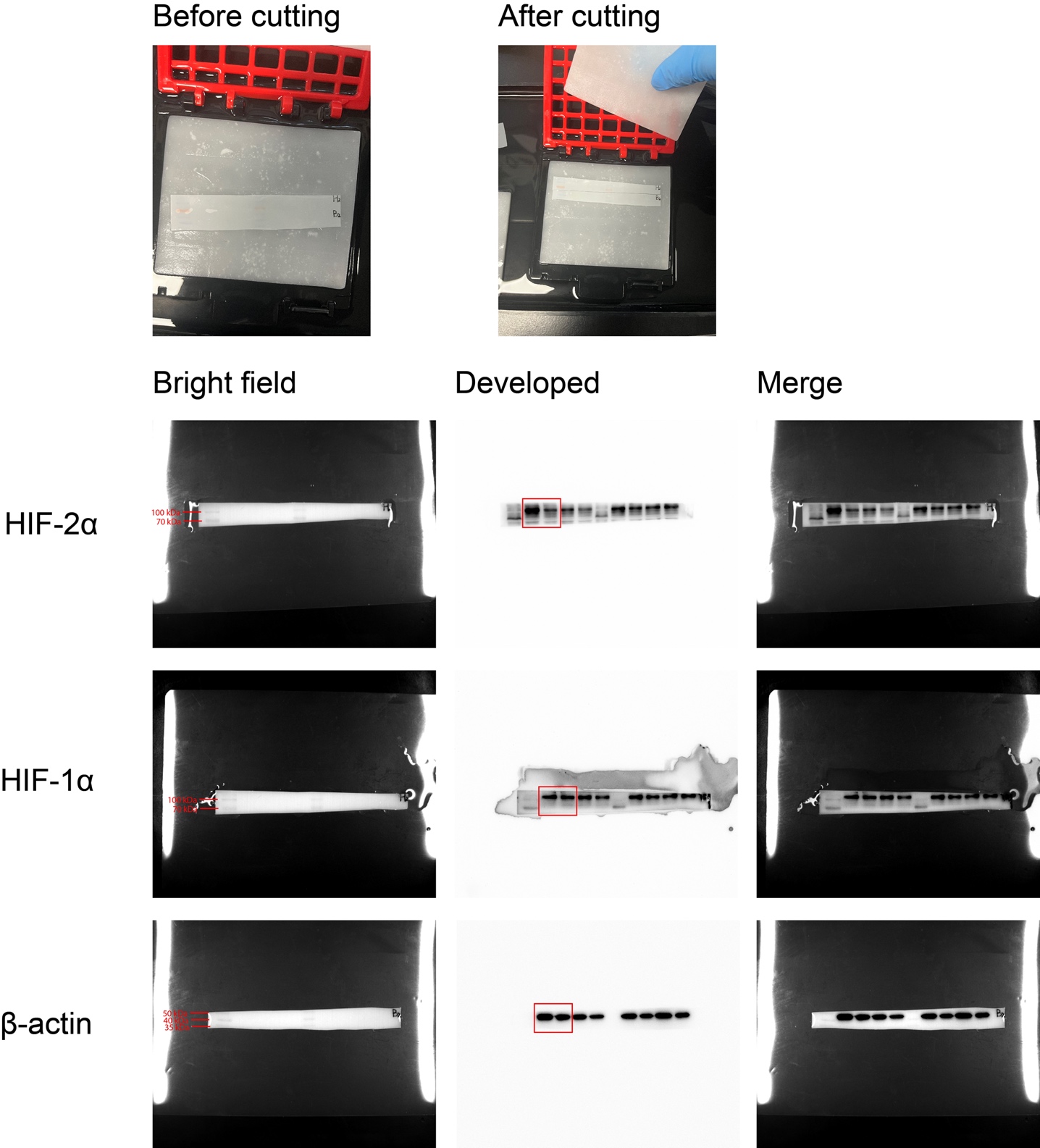


**S3 Fig. Original Western blot images corresponding to Fig 1d.** Representative uncropped blots showing HIF-2α, HIF-1α, and β-actin in HemECs treated with propranolol. The left and right sides of each blot represent two technical replicates. From left to right, lanes correspond to 0, 100, 150, and 300 µM treatment; only the 0 µM and 100 µM lanes were included in the main figure. Markers are visible to indicate approximate protein sizes.


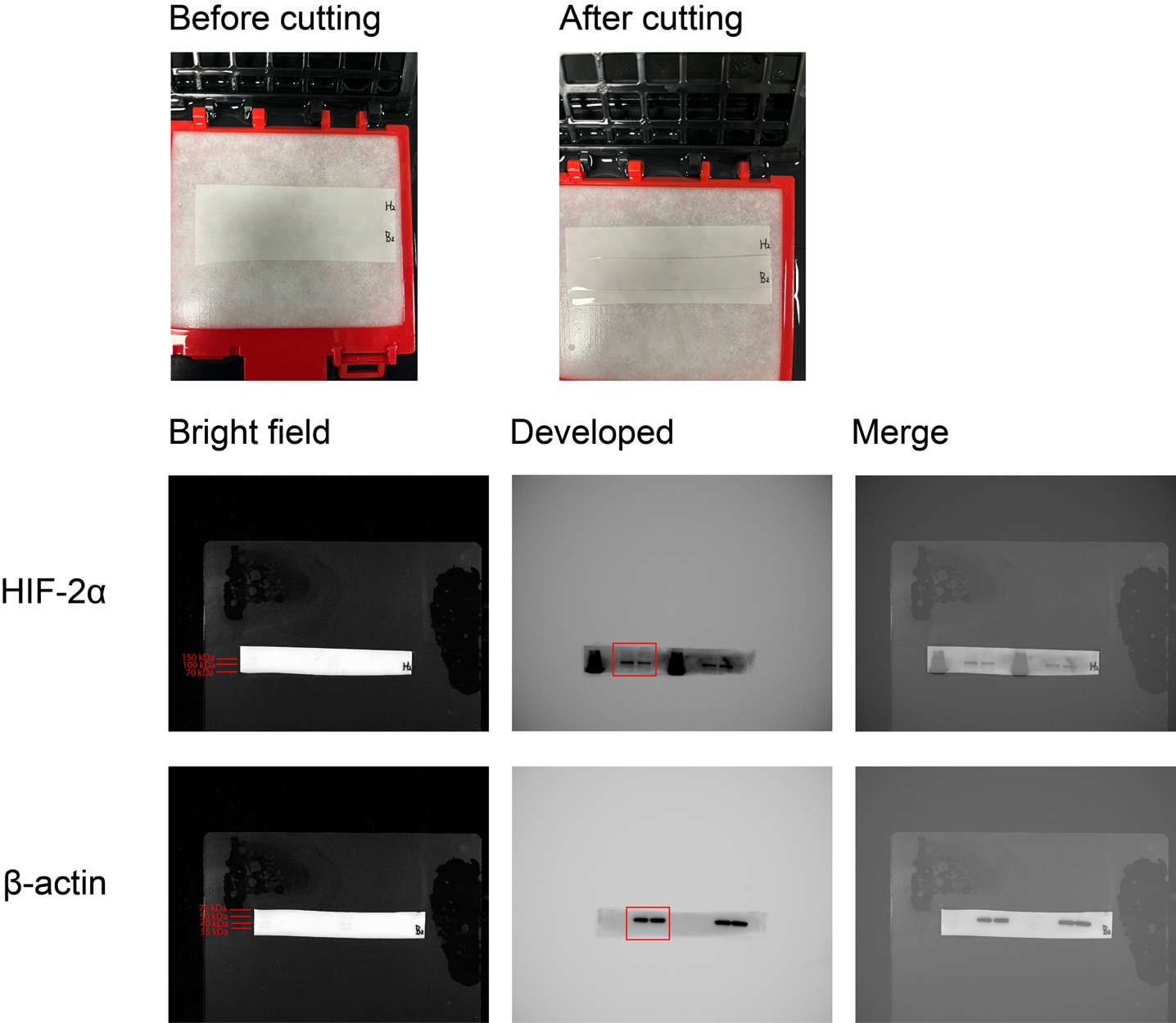


**S4 Fig.** **Original Western blot images corresponding to Fig 2c.** Representative uncropped blots showing HIF-2α and β-actin in HemECs treated with PT-2399. The left and right sides of each blot represent two technical replicates. From left to right, lanes correspond to 0 and 40 µM treatment. Markers are visible to indicate approximate protein sizes.


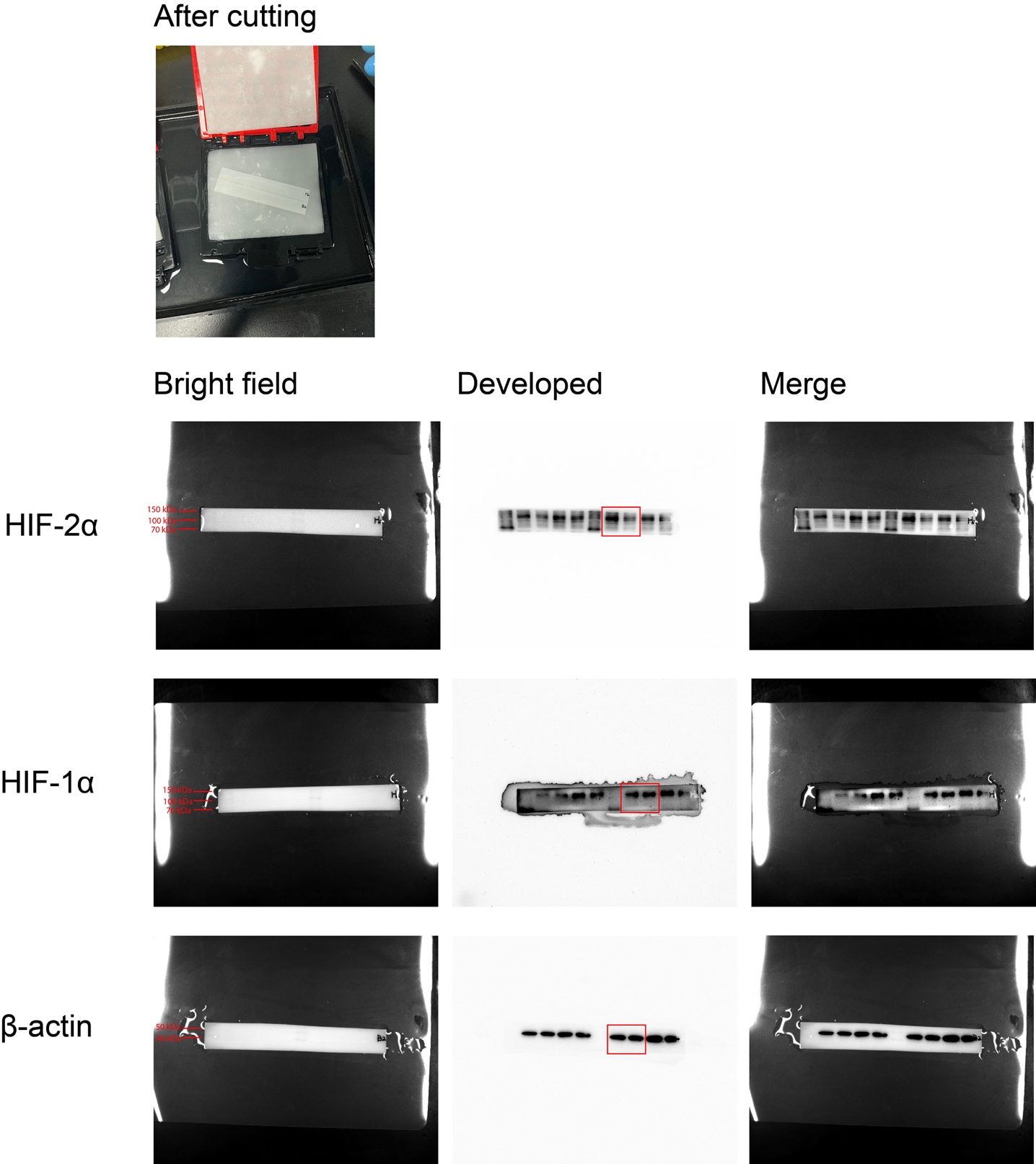


**S5 Fig. Original Western blot images corresponding to Fig 3b.** Representative uncropped blots showing HIF-2α, HIF-1α, and β-actin in sh-NC and sh-EPAS1 HemECs. The left and right sides of each blot represent two technical replicates. From left to right, lanes correspond to sh-NC, sh-EPAS1-1, sh-EPAS1-2, and sh-EPAS1-3; only the sh-NC and sh-EPAS1-1 lanes were included in the main figure. Markers are visible to indicate approximate protein sizes.

**
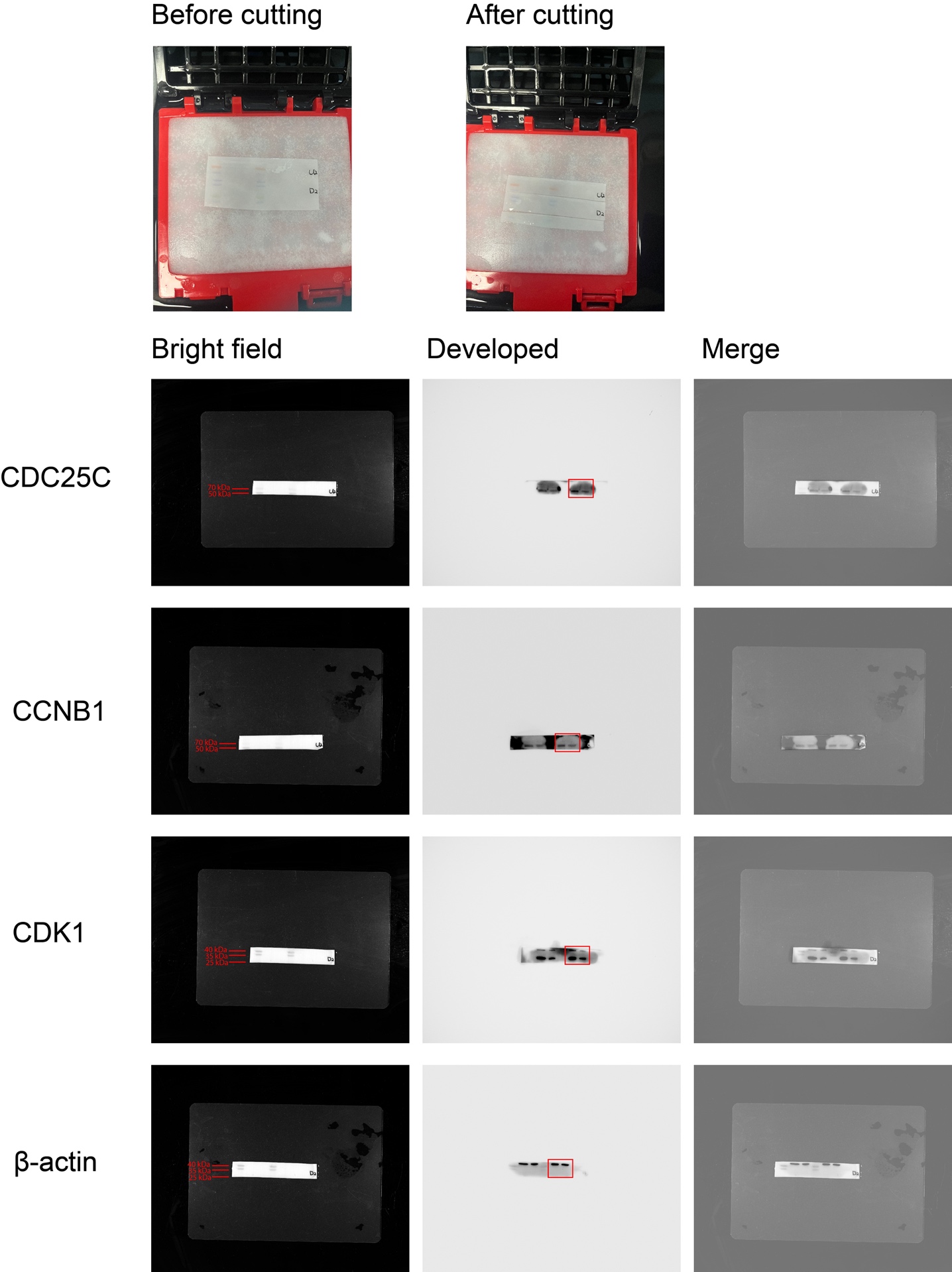
**

**S6 Fig. Original Western blot images corresponding to Fig 6a.** Representative uncropped blots showing CDC25C, CCNB1, CDK1 and β-actin in sh-NC and sh-EPAS1 HemECs. The left and right sides of each blot represent two technical replicates. Markers are visible to indicate approximate protein sizes.

**
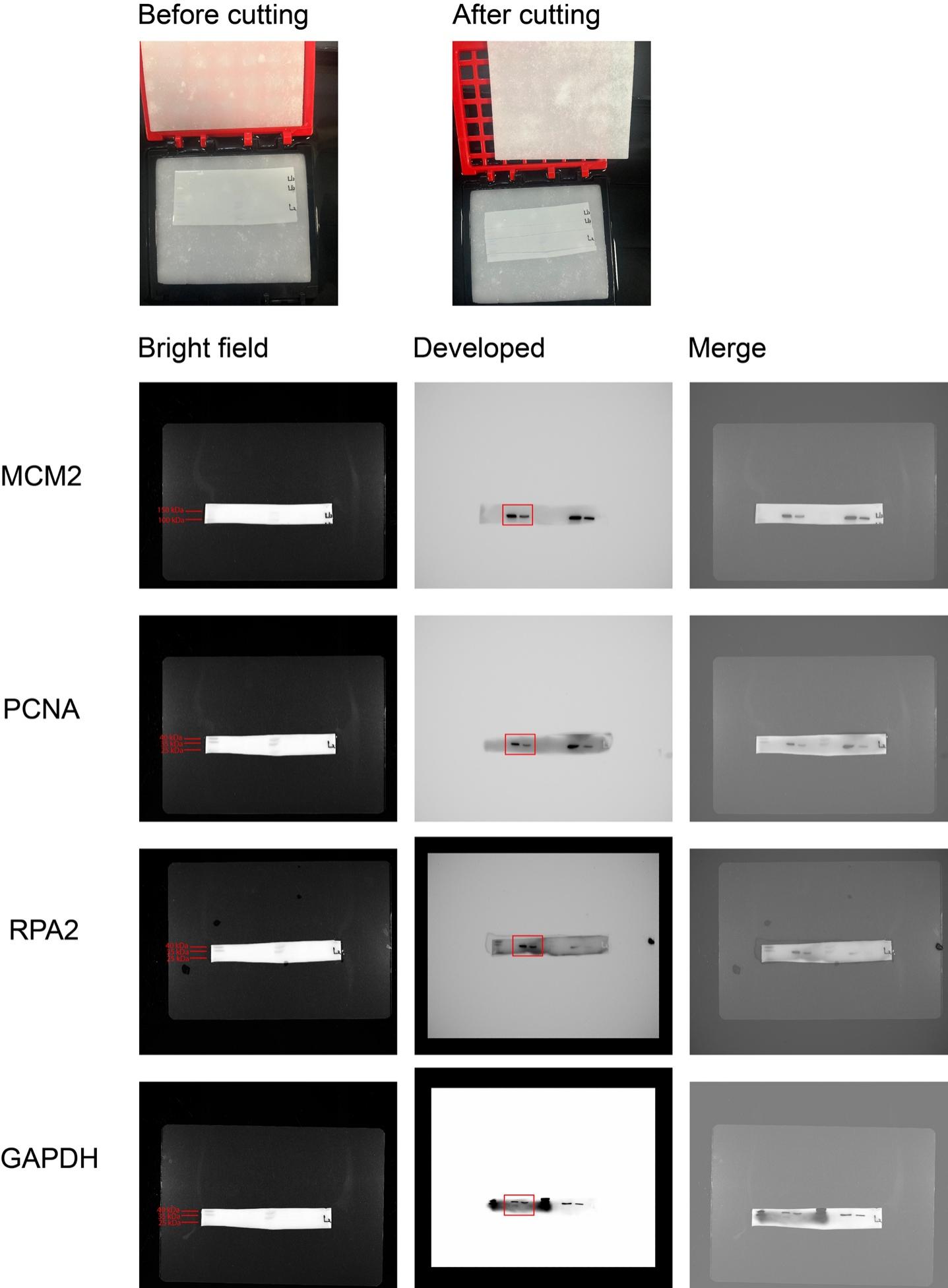
**

**S7 Fig. Original Western blot images corresponding to Fig 6c.** Representative uncropped blots showing MCM2, PCNA, RPA2 and GAPDH in sh-NC and sh-EPAS1 HemECs. The left and right sides of each blot represent two technical replicates. Markers are visible to indicate approximate protein sizes.

**
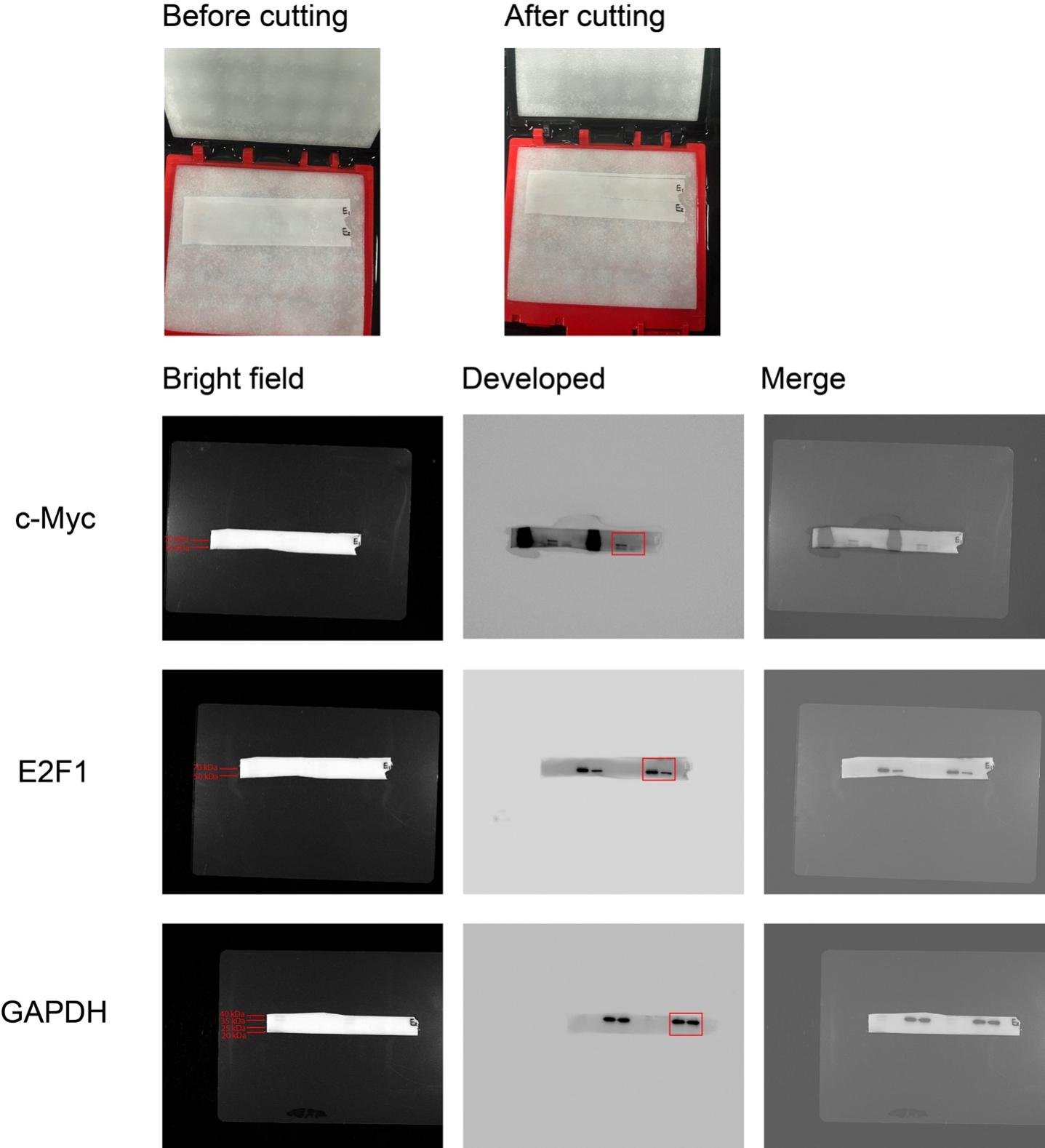
**

**S8 Fig. Original Western blot images corresponding to Fig 6e.** Representative uncropped blots showing c-Myc, E2F1 and GAPDH in sh-NC and sh-EPAS1 HemECs. The left and right sides of each blot represent two technical replicates. Markers are visible to indicate approximate protein sizes.


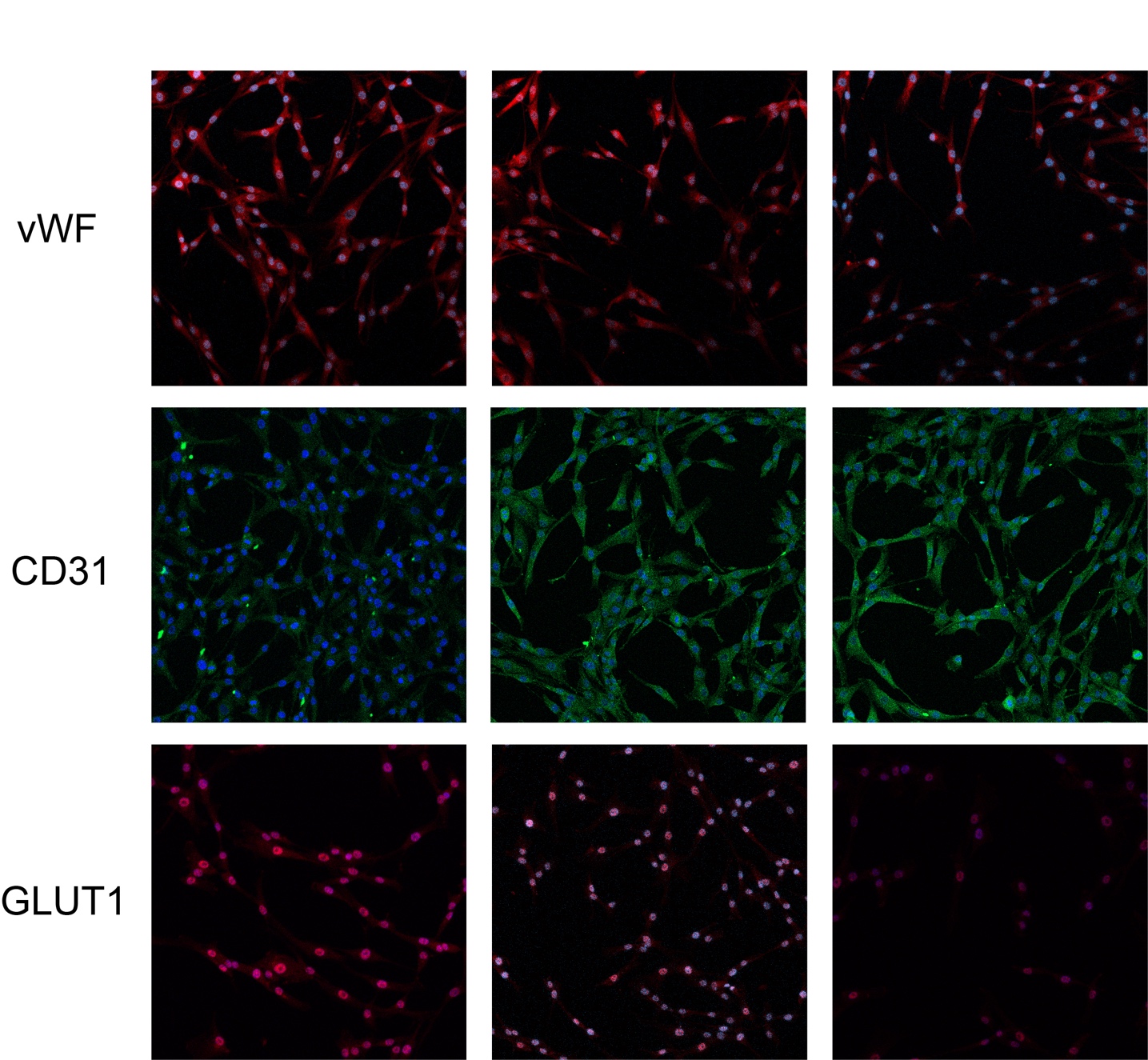


**S9 Fig. Original immunofluorescence images corresponding to Fig 1b.** Representative images of HemECs are shown. For each condition, three images are presented.

**
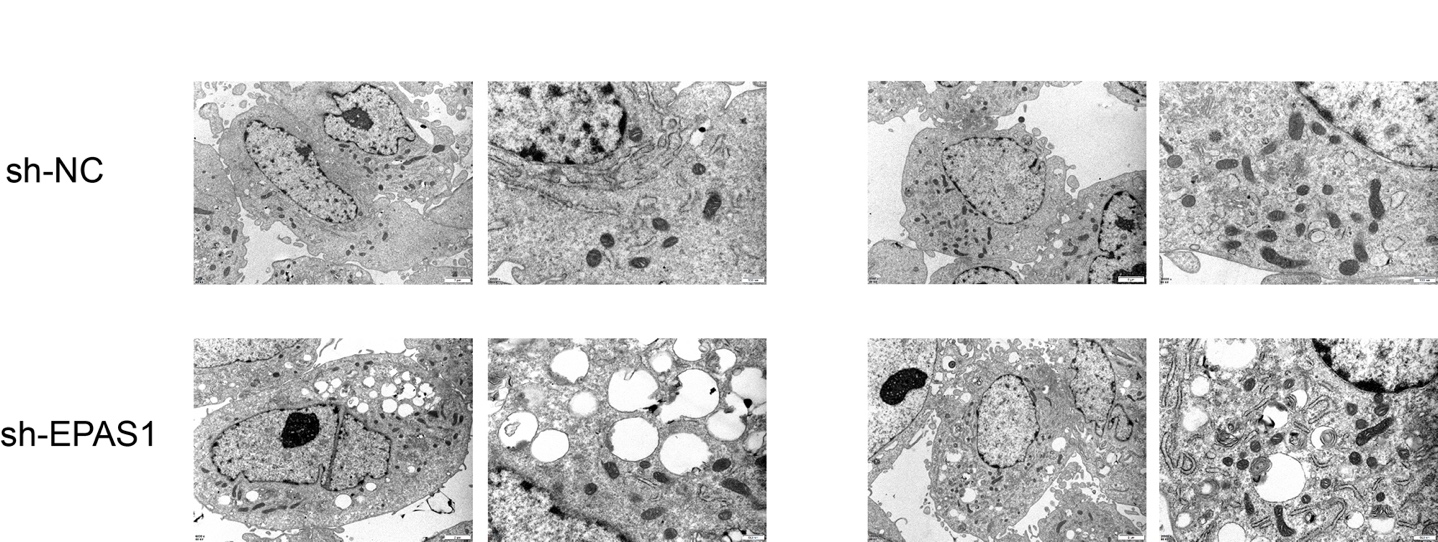
**

**S10 Fig. Original TEM images corresponding to Fig 3e.** Two additional representative TEM images from different fields of the same sample for each group are shown.

**
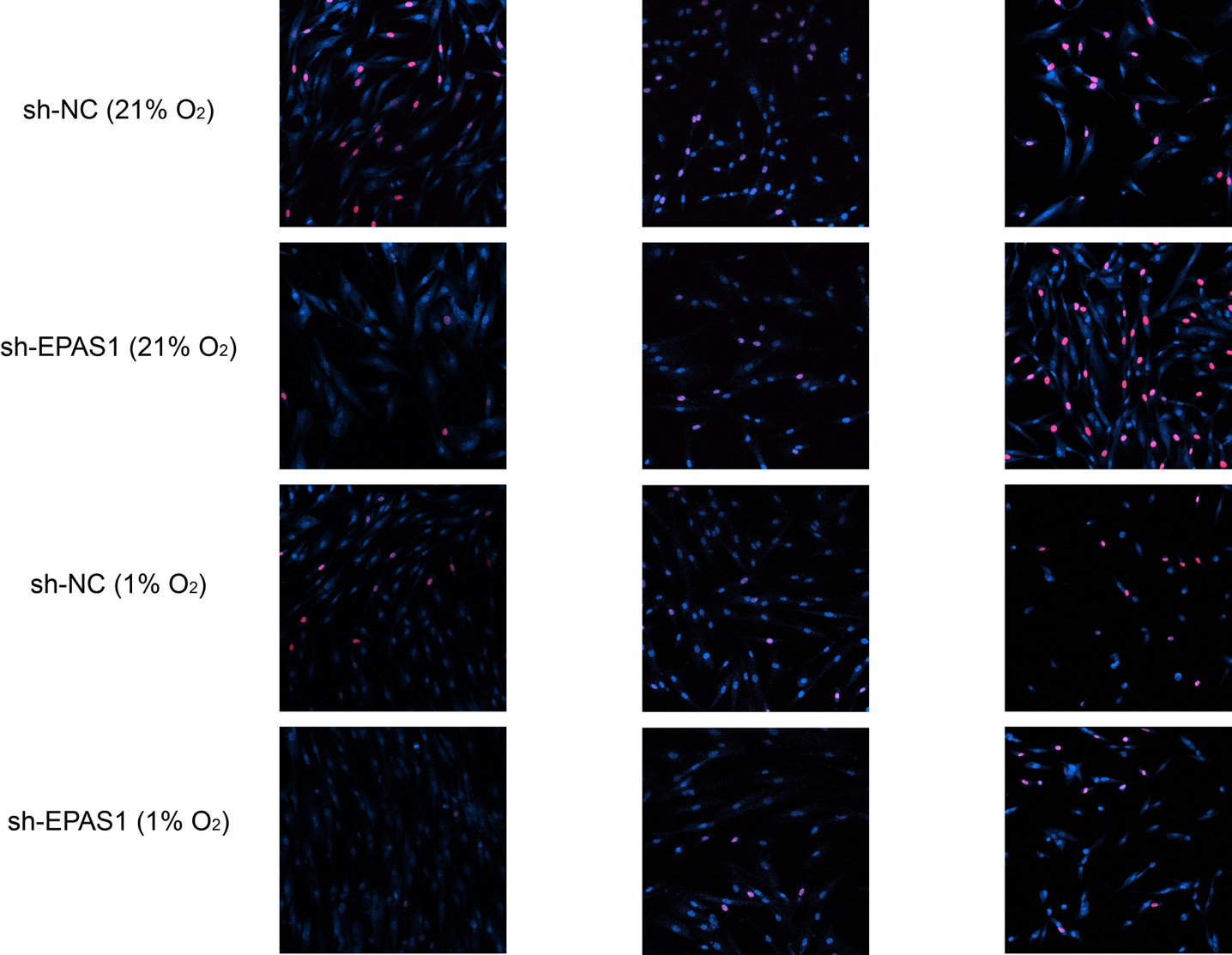
**

**S11 Fig. Original EdU staining images corresponding to Fig 4a.** Representative images of HemECs are shown. For each condition, three images are presented.


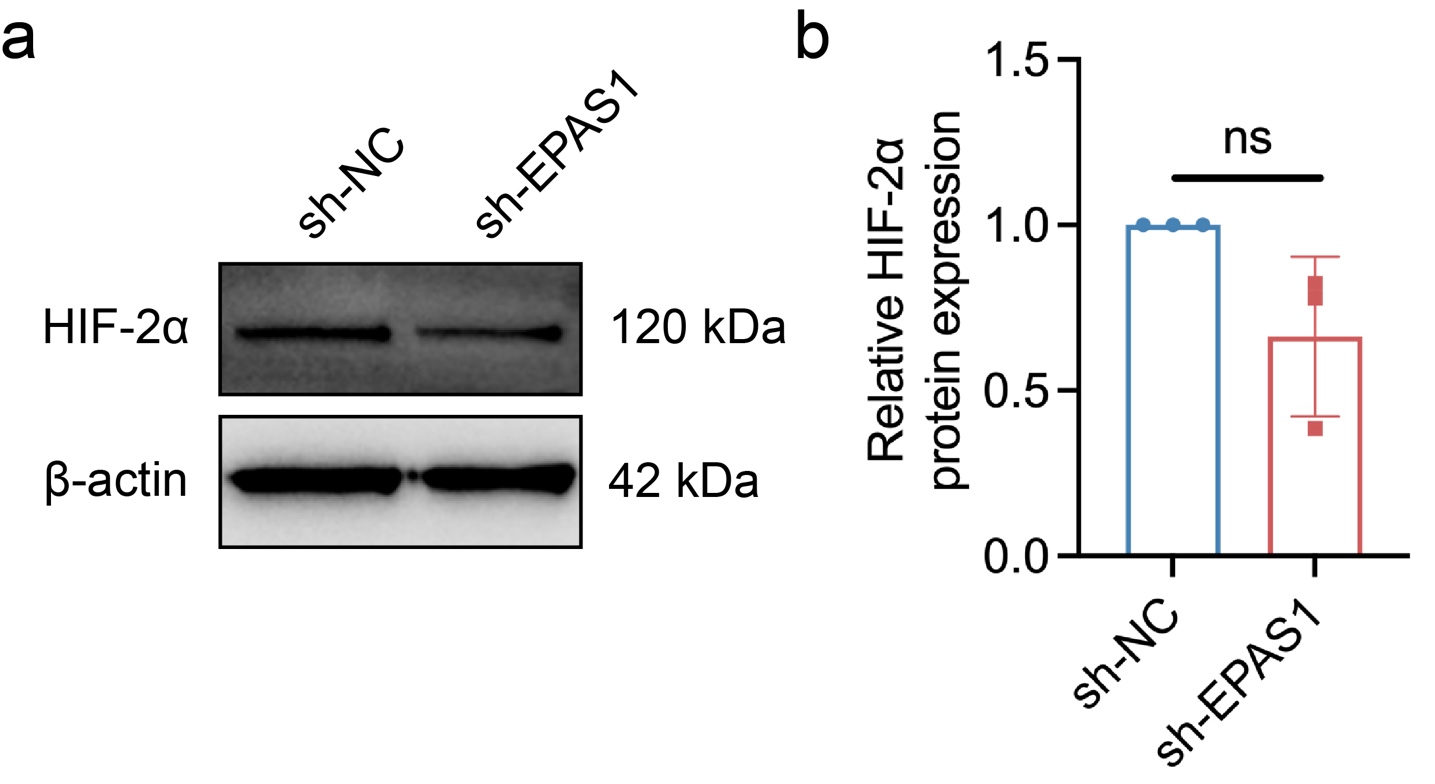


**S12 Fig. HIF-2α protein expression under normoxic conditions following *EPAS1* knockdown in HemECs.** (**a, b**) A representative Western blot image showing HIF-2α protein expression under normoxic conditions following *EPAS1* knockdown is presented in (a), with quantification shown in (b). Each data point represents an independent experimental run. Data are presented as mean ± SD. Statistical analysis was performed using each independent experimental run (*n* = 3), analyzed by a paired t-test. ns, not significant. Original blots corresponding to panel (a) are presented in S13 Fig.


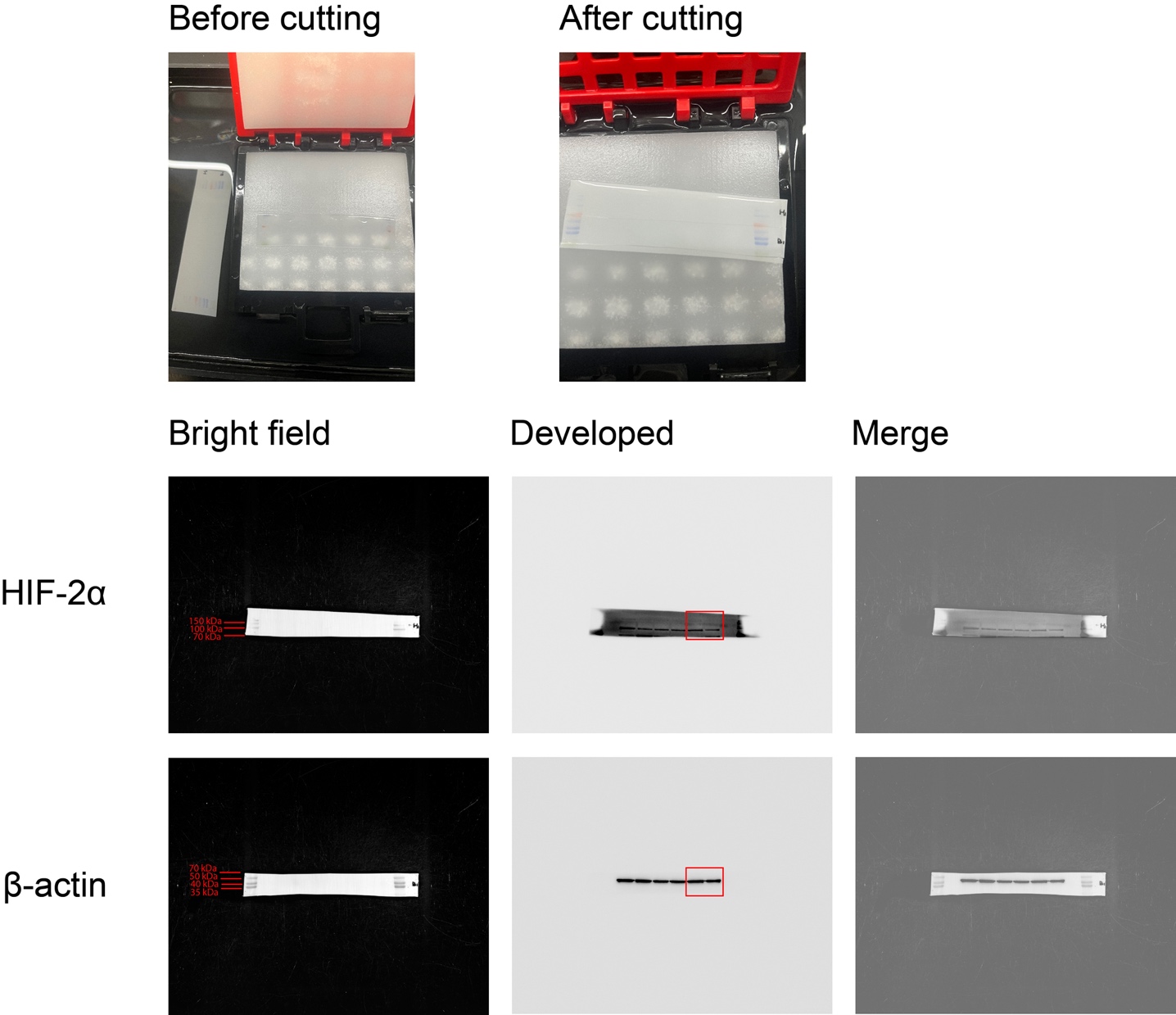


**S13 Fig. Original Western blot images corresponding to S12a Fig.** Representative uncropped blots showing HIF-2α and β-actin in sh-NC and sh-EPAS1 HemECs. Each blot represents three independent experimental runs. Markers are visible to indicate approximate protein sizes.
