# Supplementary material for: EPAS1 knockdown is associated with cell cycle and DNA replication programs and MYC/E2F-related signatures in hemangioma endothelial cells
Source: PLoS One. 2026 Jul 24;21(7):e0354272. doi: 10.1371/journal.pone.0354272 (PMC13399520; doi:10.1371/journal.pone.0354272)
Supplement: S2 File — (PDF) [file pone.0354272.s002.pdf]

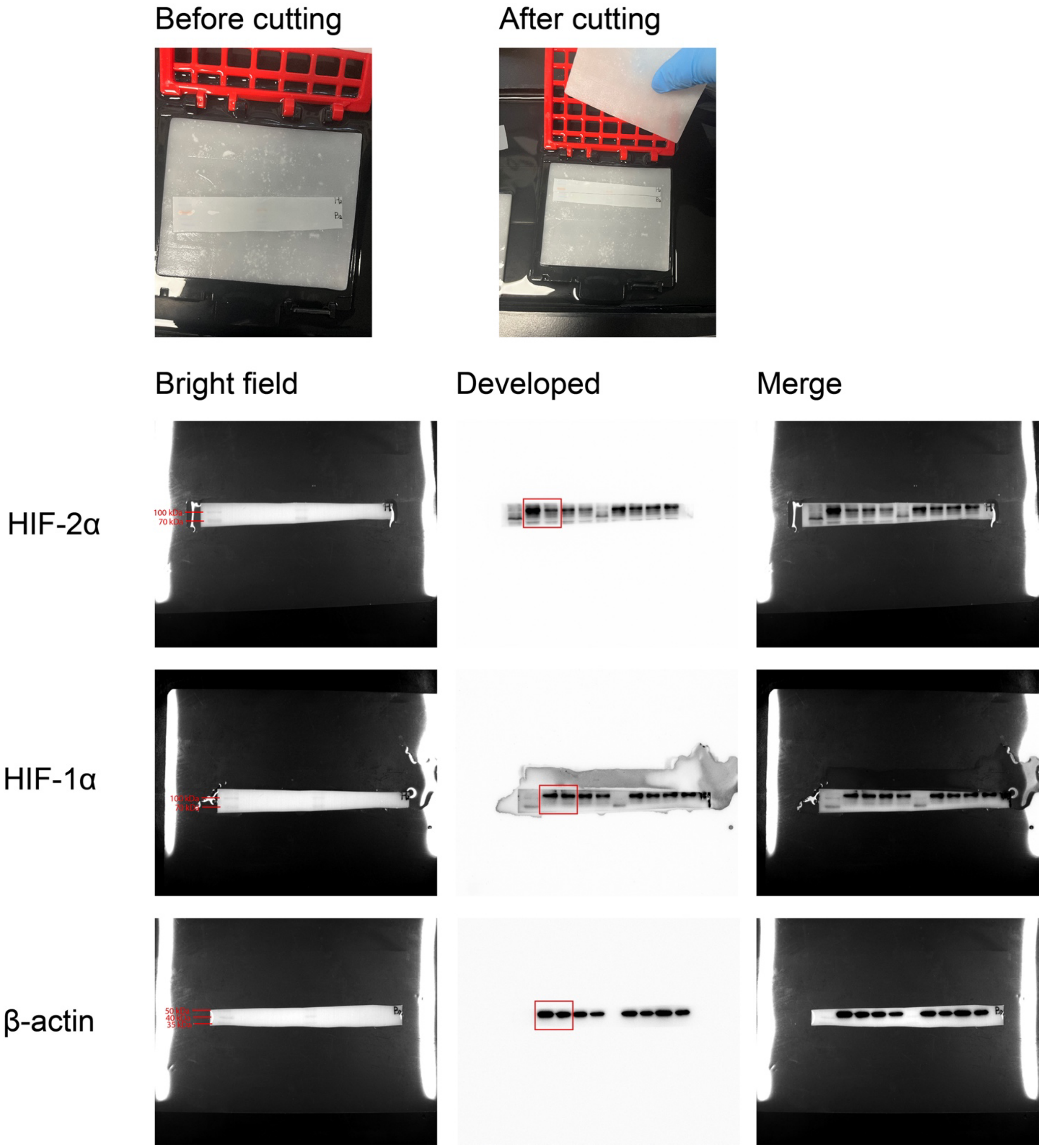

S3 Fig. Original Western blot images corresponding to Fig 1d. Representative uncropped blots showing HIF-2α, HIF-1α, and β-actin in HemECs treated with propranolol. The left and right sides of each blot represent two technical replicates. From left to right, lanes correspond to

0, 100, 150, and 300 μM treatment; only the 0 μM and 100 μM lanes were included in the main figure. Markers are visible to indicate approximate protein sizes.

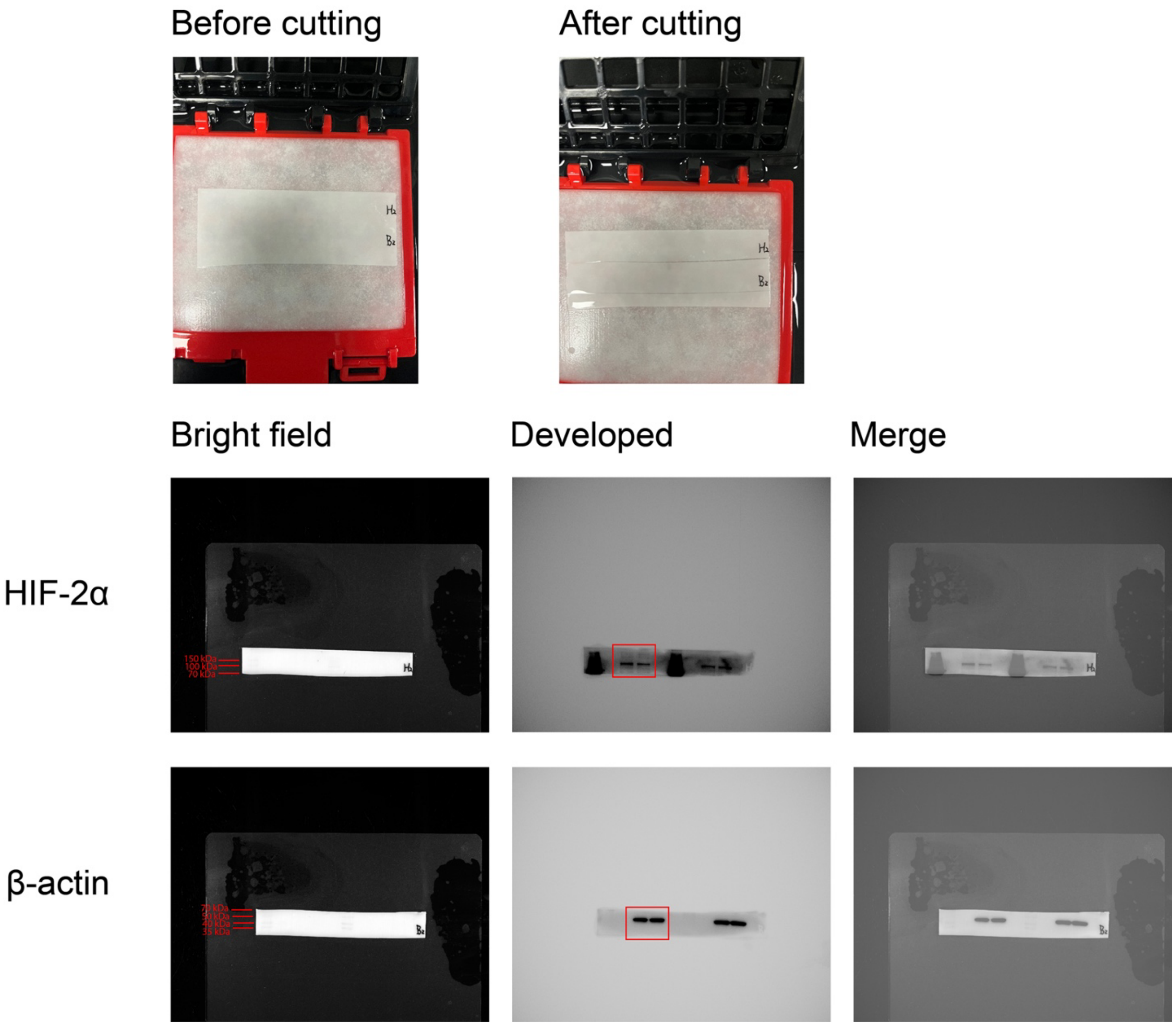

S4 Fig. Original Western blot images corresponding to Fig 2c. Representative uncropped blots showing HIF-2α and β-actin in HemECs treated with PT-2399. The left and right sides of each blot represent two technical replicates. From left to right, lanes correspond to 0 and 40 μM treatment. Markers are visible to indicate approximate protein sizes.

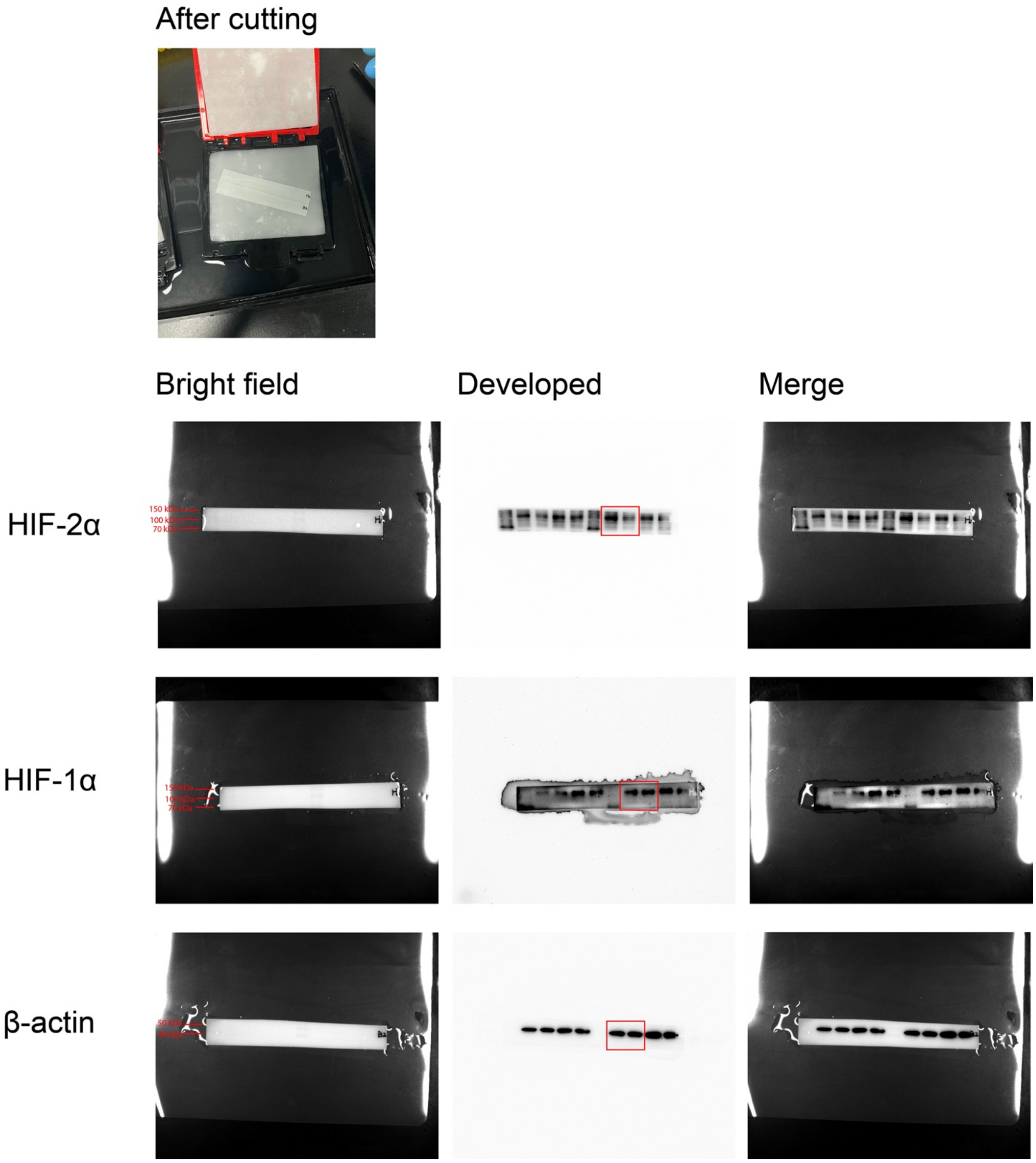

S5 Fig. Original Western blot images corresponding to Fig 3b. Representative uncropped blots showing HIF-2α, HIF-1α, and β-actin in sh-NC and sh-EPAS1 HemECs. The left and right sides of each blot represent two technical replicates. From left to right, lanes correspond to sh-

NC, sh-EPAS1-1, sh-EPAS1-2, and sh-EPAS1-3; only the sh-NC and sh-EPAS1-1 lanes were included in the main figure. Markers are visible to indicate approximate protein sizes.

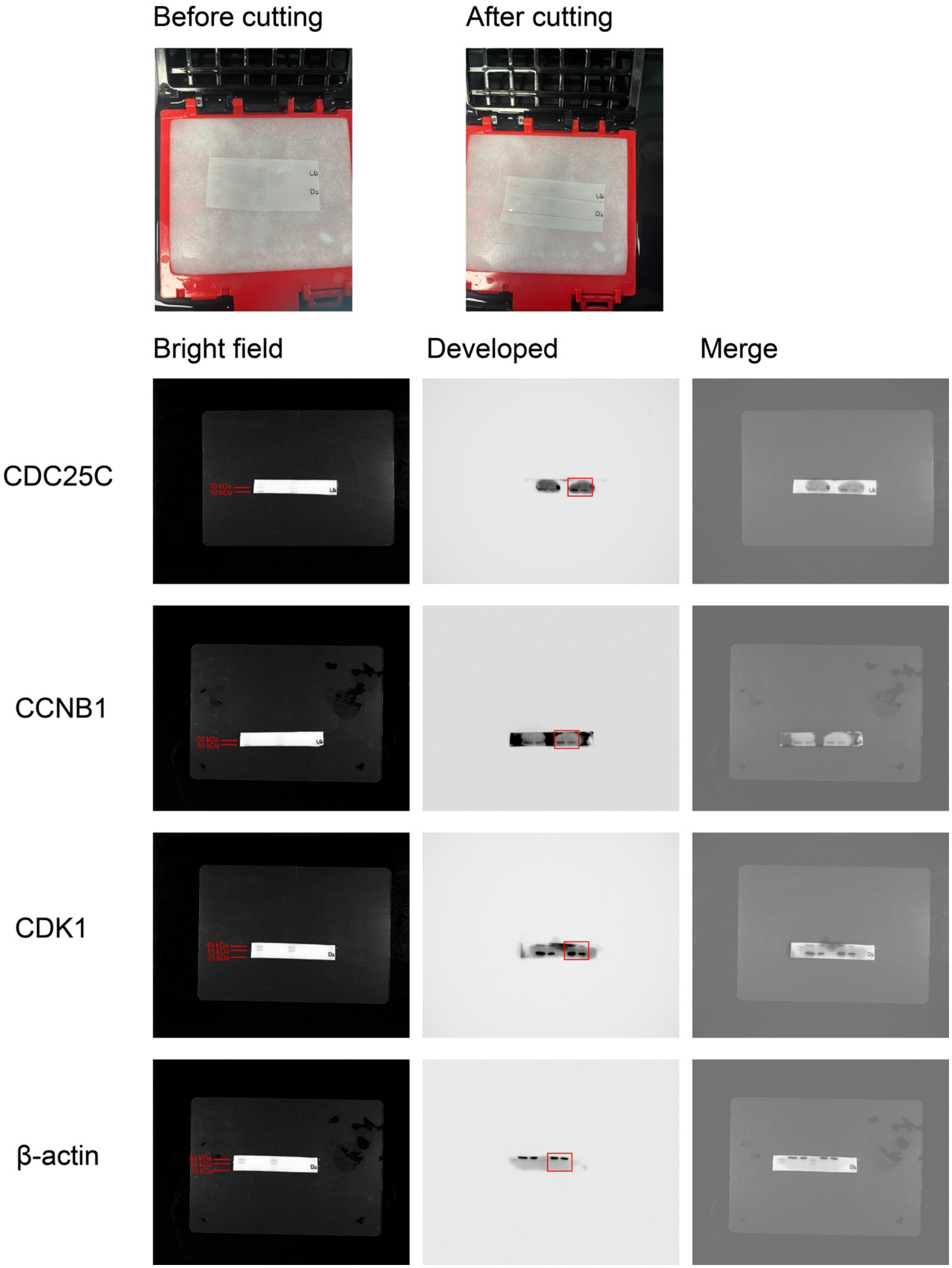
Before cutting
After cutting
Bright field
Developed
Merge
CDC25C
70 kDa
50 kDa
CCNB1
70 kDa
50 kDa
CDK1
40 kDa
35 kDa
25 kDa
β-actin
40 kDa
35 kDa
25 kDa

S6 Fig. Original Western blot images corresponding to Fig 6a. Representative uncropped blots showing CDC25C, CCNB1, CDK1 and β-actin in sh-NC and sh-EPAS1 HemECs. The left and right sides of each blot represent two technical replicates. Markers are visible to indicate approximate protein sizes.

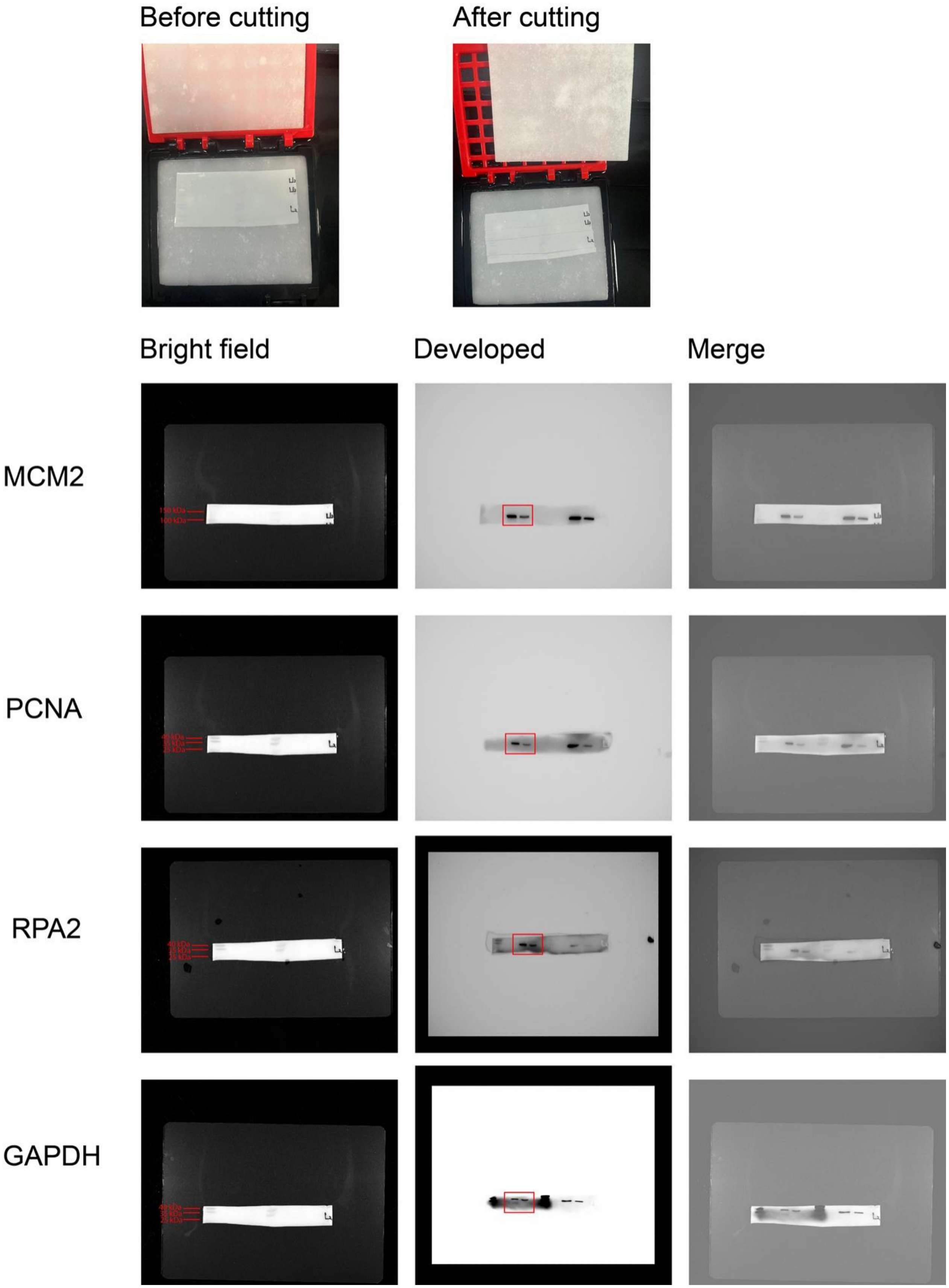
Before cutting
After cutting
Bright field
Developed
Merge
MCM2
PCNA
RPA2
GAPDH

S7 Fig. Original Western blot images corresponding to Fig 6c. Representative uncropped blots showing MCM2, PCNA, RPA2 and GAPDH in sh-NC and sh-EPAS1 HemECs. The left and right sides of each blot represent two technical replicates. Markers are visible to indicate approximate protein sizes.

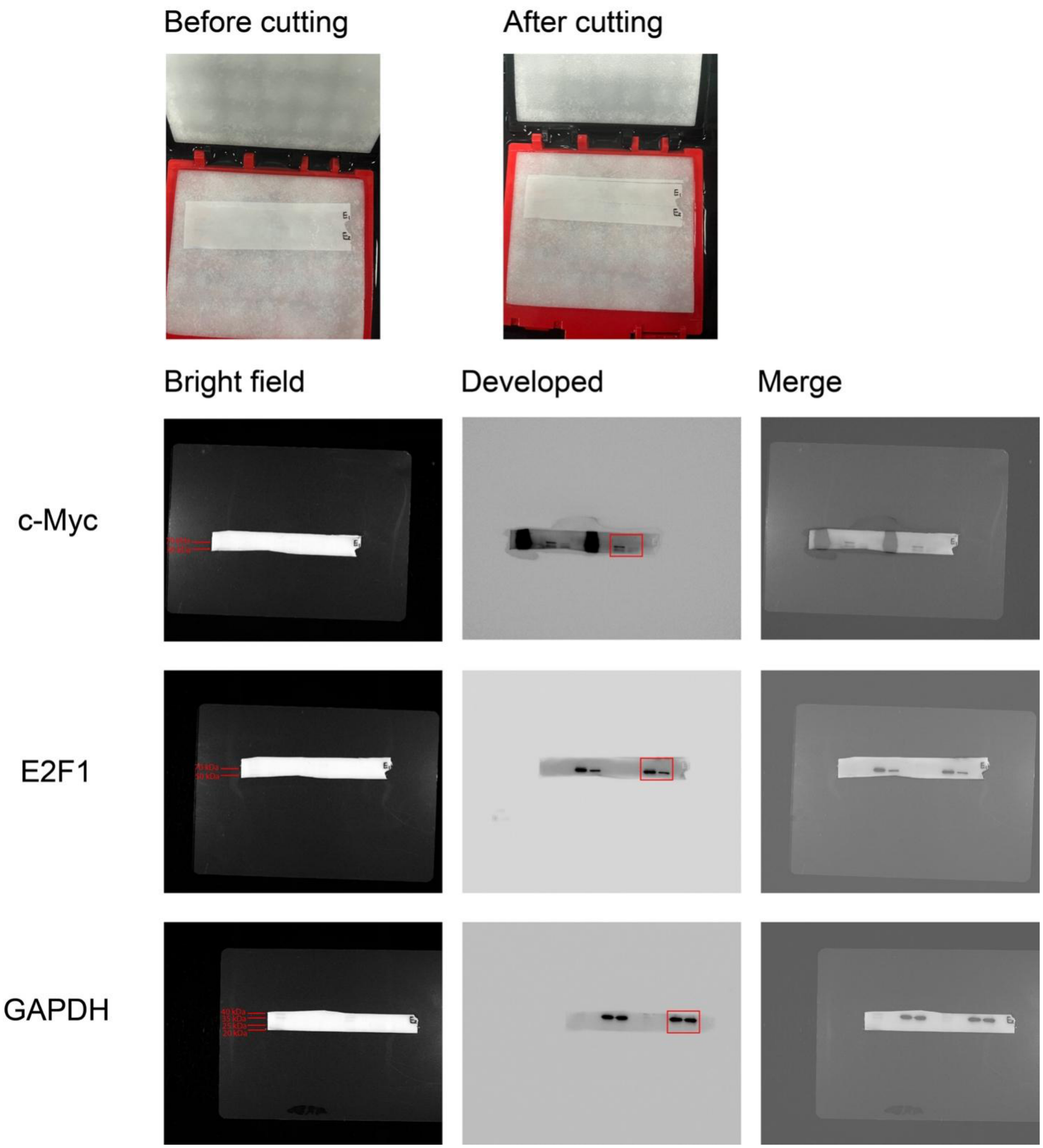

S8 Fig. Original Western blot images corresponding to Fig 6e. Representative uncropped blots showing c-Myc, E2F1 and GAPDH in sh-NC and sh-EPAS1 HemECs. The left and right sides of each blot represent two technical replicates. Markers are visible to indicate approximate protein sizes.

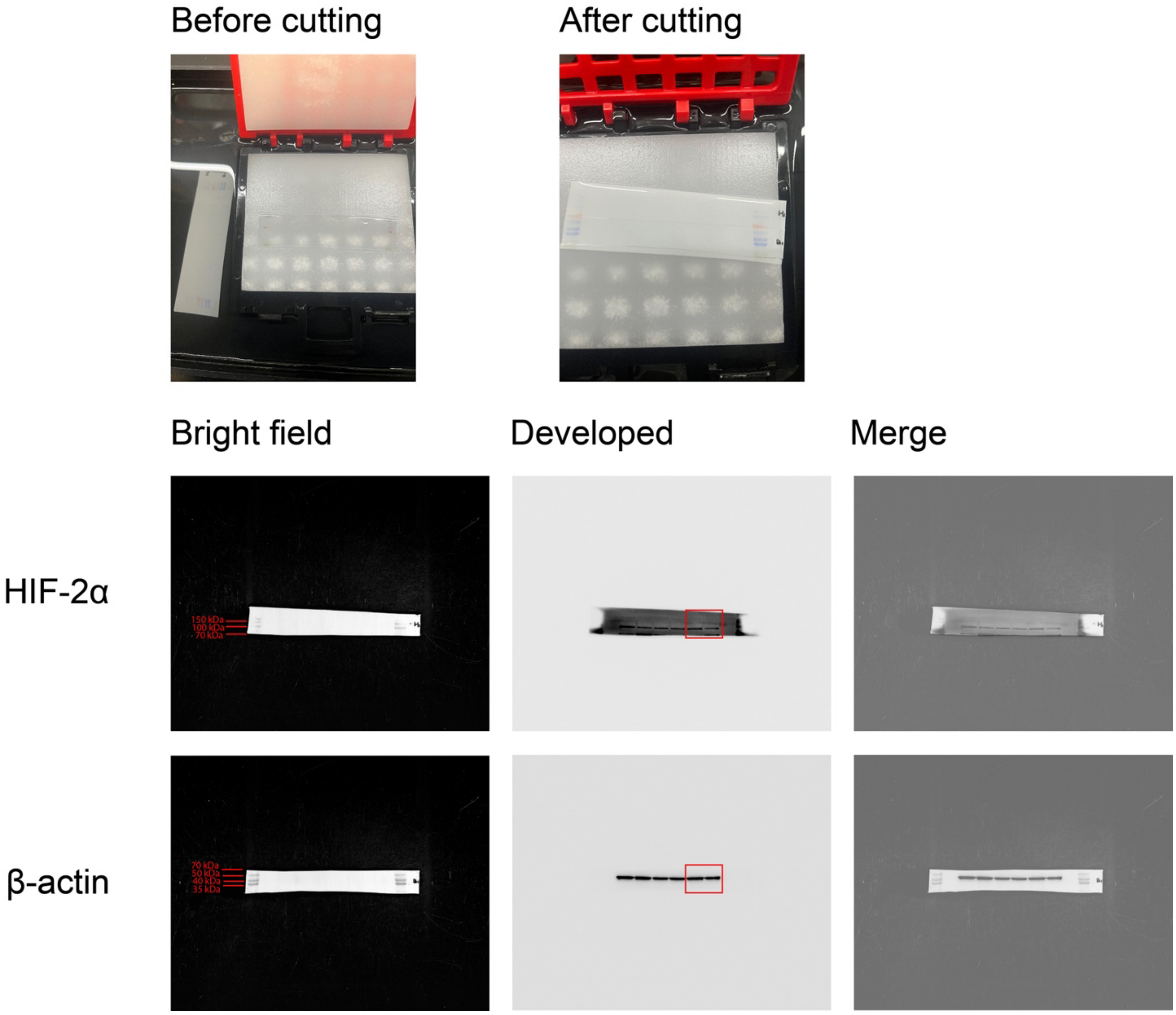

S13 Fig. Original Western blot images corresponding to S12a Fig. Representative uncropped blots showing HIF-2α and β-actin in sh-NC and sh-EPAS1 HemECs. Each blot represents three independent experimental runs. Markers are visible to indicate approximate protein sizes.
